# Endophyte Inoculation and Elevated Potassium Supply on Productivity, Growth and Physiological Parameters of Spring Barley (*Hordeum vulgare* L.) Genotypes over Contrasting Seasons

**DOI:** 10.3390/plants13081168

**Published:** 2024-04-22

**Authors:** Dominik Bleša, Pavel Matušinský, Milan Baláž, Zdeněk Nesvadba, Marta Zavřelová

**Affiliations:** 1 Agrotest Fyto, Ltd., 76701 Kroměříž, Czech Republic; 2 Department of Experimental Biology, Faculty of Science, Masaryk University, 62500 Brno, Czech Republic; balaz@sci.muni.cz; 3 Department of Botany, Faculty of Science, Palacký University in Olomouc, 78371 Olomouc, Czech Republic; 4 Gene Bank, Crop Research Institute, Drnovská 507, 16106 Praha 6 – Ruzyně, Czech Republic; nesvadba@vurv.cz

**Keywords:** spring barley, genotype, *Serendipita indica*, potassium, carbon stable isotope, δ13C, soluble sugars, drought

## Abstract

In recent years, recurrent droughts have significantly affected spring barley production, reducing the quantity and quality of grain. This study aims to identify genotype-specific traits and the drought resilience of six different *Hordeum vulgare* L. (spring barley) genotypes, while also examining the potential of potassium application and fungal endophyte *Serendipita indica* inoculation to mitigate the negative effects of dry periods during the growing season. Field experiments were conducted over a three-year period from 2020 to 2022, measuring physiological, growth, and yield parameters. To get insight into the physiological state of the plants, we measured the soluble sugars content and the ratio of stable carbon isotopes in the flag leaf tissue, which reflects conditions during its formation. The dominant factors that influenced the measured parameters were the genotypes and seasons, as well as their interaction, rather than other experimental factors. The results showed that the Spitfire and Accordine varieties were the best performing in both the 2020 and 2021 seasons, as indicated by their yield. However, in the drier 2022 season, the yield of these two varieties decreased significantly (to 55% for Spitfire and to 69% for Accordine of their yield in 2021), while for the arid-region genotypes, it remained at the same level as the previous year. This study sheds light on the potential of various genotypes to withstand periods of drought and the effectiveness of using potassium application and *S. indica* inoculation as mitigation approaches.

## 1. Introduction

As the climate undergoes significant changes in precipitation patterns, plants are exposed to longer periods of water scarcity [1]. This lack of water can lead to a range of problems, including nutrient uptake limitation, decreased turgor with mechanical consequences, reduced growth rate and biomass accumulation, hormonal imbalances, altered cell division and proliferation, and limitations in photosynthetic processes due to limited gas exchange [2,3]. Drought susceptibility can be influenced by a range of factors, including environmental conditions such as soil quality, nutrient supply, and the biotic components of the ecosystem, as well as agronomic interventions [4,5]. Long-term exposure to drought can also cause soil degradation, exacerbating the problem [6].

Despite these challenges, plants have developed numerous strategies to defend themselves against drought stress, including anatomical and morphological changes, as well as biochemical and physical mechanisms [7,8,9]. These changes can either be adaptations, genetic changes that occur over many generations, or acclimation responses that occur during an individual organism’s lifetime [10,11]. Symbiotic associations with bacteria or fungi, such as mycorrhizal symbioses or endophytic life strategies, have coevolved with plants and can support adaptation and acclimatization processes [12,13]. Symbiotic microorganisms can mediate the transport of nutrients to the roots, maintain a favorable environment in the soil, modulate the stomatal opening or accumulation of osmotically active substances in plant tissues [14,15], or prevent pathogenic species from attacking weakened plants [16,17]. *Serendipita indica* is a potential plant growth-promoting fungus, that has been found to improve crop production under abiotic stress conditions [18,19]. This symbiotic fungus can modulate barley morphology and physiology, and change plant metabolic activities, such as CO_2_ assimilation and transpiration rate [20,21,22,23,24]. It is important to understand the various factors that can influence a plant’s responses to drought and the potential role of symbiotic organisms in mitigating the negative effects of water scarcity, as the knowledge of them is so far incomplete.

The quest for crop protection and the stabilization of food self-sufficiency and sustainability has led to various practical approaches, including breeding and the use of resistant varieties or genetic resources from regions with drier climate. Comparing these genotypes can help identify the best options for crop production under drought stress [25,26]. In addition to breeding, appropriate agrotechnical soil preparation can also play a significant role in reducing the impact of drought stress on plants. It can delay stress onset or mitigate the effects of associated stresses, thus ensuring healthy crop growth [15,27].

Nutrient availability is another crucial factor influencing crop health, yield, and quality. To alleviate the negative effects of drought stress, large doses of fertilizers are often applied. Appropriate timing of application is essential to ensure that plants have sufficient nutrients for growth and to maintain osmotic potential in their tissues [28]. This is crucial for proper water management through stomatal regulation [29,30]. Potassium is a basic element in helping plants overcome the effects of drought. It affects various developmental processes during drought periods, including increasing the osmotic pressure in the roots and enhancing water uptake from the environment [31,32,33]. It is also essential for fruit (grain) formation and the regulation of stomatal conductivity. Potassium plays a critical role in cytoplasmic pH, the regulation of membrane potential, and enzymatic activity [31,34,35]. Therefore, adequate potassium supply is crucial in mitigating the negative effects of drought stress on crop productivity and overall postharvest quality [36].

To comprehensively assess the drought tolerance of plants, various approaches are available, including non-destructive techniques such as fluorescence curves or spectral indexes, as well as destructive methods, such as the analysis of primary and secondary metabolites or the evaluation of plant organ weight [11,37]. However, overall approaches considering growth characteristics, yield, and product quality are usually preferred in agro-systems, even though these can be influenced by other factors [38,39]. To gain insight into the functionality of the plant’s assimilation apparatus, the soluble sugars content can be measured. Under water stress, tolerant barley genotypes increase the accumulation of soluble sugars as osmoprotectants, which protect the cells from damage [40].

Another useful characteristic for evaluating drought stress is the ratio of stable carbon isotopes present in plant tissues. The shift in the carbon isotope ratio indicates long-term changes in gas flow through the stomata, regulating water management. Reduced water availability causes stomatal closure and consequently enhances stable carbon isotope ratio [11,41]. Moreover, this ratio is also linked to water use efficiency and can be utilized to indirectly assess plant acclimation under drought-stress conditions [42,43]. By incorporating these techniques and characteristics, a more comprehensive evaluation of drought tolerance in plants can be achieved.

While assessing the drought tolerance of barley, there are various methods that can be used, but, ultimately, the crop yield should be the key factor to consider, as it directly affects the economic value of the harvest. The total plant biomass or water use efficiency may not provide a comprehensive evaluation of genotypes for stress tolerance; however, together with other parameters such as plant height, leaf length and width, and the number of productive tillers per m^2^, they should also be considered, as they can indicate the potential productivity of the plant [44].

This study hypothesizes that potassium supplementation and *S. indica* inoculation significantly enhances the drought resilience and productivity of spring barley genotypes adapted to local conditions, as well as those originating from arid regions. While previous studies have explored the individual effects of potassium and endophytes on plant stress responses [14,15,21,22,28,29,30,31], their combined impact on various spring barley genotypes, particularly under field conditions, remains insufficiently understood. By investigating genotype-specific traits associated with drought resilience and evaluating the effects of these interventions on productivity and growth parameters, the study aims to offer insights into strategies for stabilizing crop production under non-standard climatic conditions.

In this article, we aimed to measure the productivity (yield, weight of thousand grains—TGW—and starch content in grains) of six genotypes of spring barley genotypes under natural field conditions during three cultivation seasons and the possibility of increasing their productivity with potassium fertilizer and inoculation with *S. indica*. We also assessed genotype-specific traits, such as leaf length and width, plant height, and the number of productive tillers per m^2^. We also aimed to assess the response of spring barley genotypes in the early stages of plant development to short-term drought.

## 2. Materials and Methods

### 2.1. Description of the Experimental Field Site and Design

In completely randomized factorial designs, experiments were conducted over three years (2020–2022) in 2.5 m^2^ plots in the field at the Agriculture Research Institute in Kroměříž, Czech Republic. The experiment was conducted across non-contiguous locations with the application of endophyte and potassium fertilizer each season. The experimental factors were three potassium fertilizer (KCl) rates, 0, 100, and 200 kg/ha, six spring barley genotypes from the Agriculture Research Institute in Kroměříž gene bank (Sebastian, Spitfire, Accordine, Nutans Afganistan, CPI 18197, and CI 6388), and *S. indica*-uninoculated (NI), and *S. indica*-inoculated treatments.

Characteristics of cultivars are available in GRIN Czech database under the following accession numbers: Sebastian, 03C0602773; Spitfire, 03C0603158; Accordine, 03C0603157; Nutans Afganistan, 03C0600984; CPI 18197, 03C0602165; and CI 6388, 03C0602060. The malting barley varieties Spitfire and Accordine were bred in the Czech Republic and Germany, respectively, and were registered in 2018. The Sebastian variety originates from Denmark and was registered in the Czech Republic in 2005. These varieties represent relatively new cultivars specifically adapted to local climate conditions, with breeding efforts focused on achieving high malting quality and yield. In contrast, Nutans Afganistan is a genetic resource originating from Afghanistan, which has been part of the National Gene Bank collection in the Czech Republic since 1958. CPI 18197 is another genetic resource, originating from Algeria, acquired by the Australian research organization CSIRO in 1962 during a collecting expedition. CI 6388, originating from Ethiopia, was acquired in 1938 for the National Small Grains Collection in the USA.

Sebastian, Spitfire, and Accordine were chosen for their high yield potential and malting quality, reflecting current agricultural production priorities. Nutans Afganistan, CPI 18197, and CI 6388 might possess unique adaptations enabling them to withstand water scarcity, offering valuable genes for breeding programs aimed at enhancing drought resilience.

**Figure 1:**
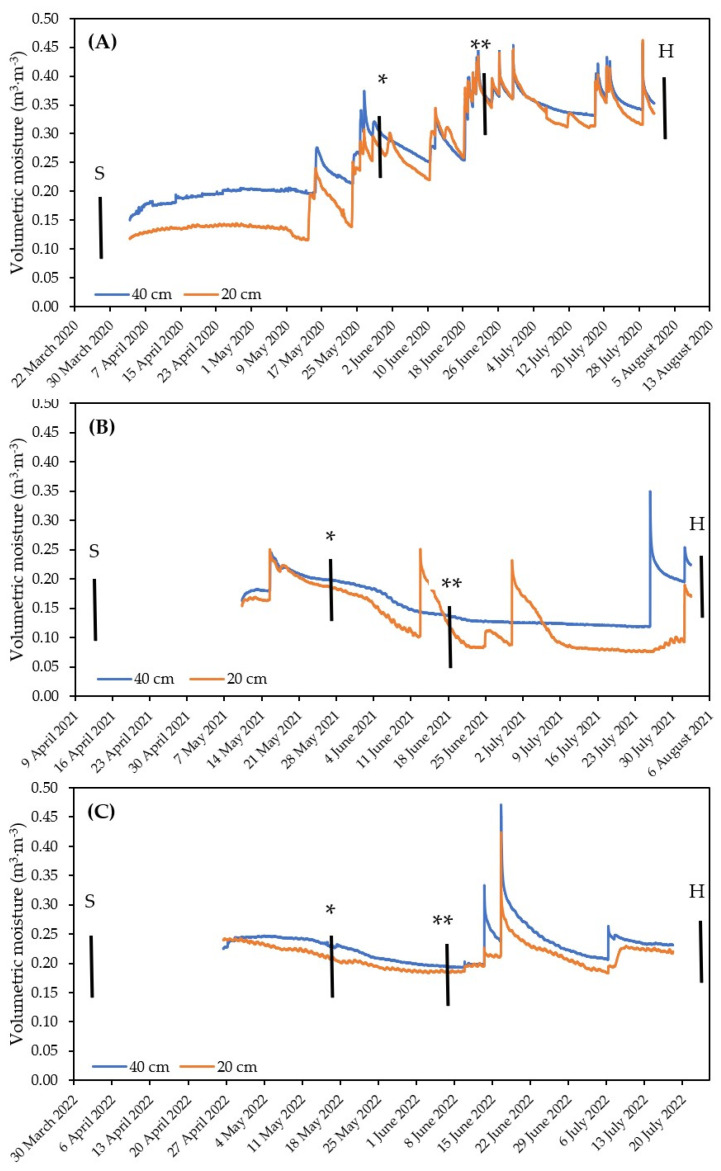
Volumetric soil moisture at 20 and 40 cm under the soil surface in the spring seasons: (**A**) 2020, (**B**) 2021, (**C**) 2022. S—sowing; H—harvesting; *—soluble sugars I sampling; **—soluble sugars II sampling.

The field location had an altitude of up to 220 m above sea level; in 2020, it had a temperature of 10.5 °C, annual rainfall of 776 mm, and rainfall of 370 mm during the growing season. In 2021, the annual temperature was 9.6 °C, annual rainfall 545 mm, and rainfall 175 mm during the growing season. In 2022, the annual temperature was 10.7 °C, the rainfall 479 mm, and, during the growing season, 184 mm (Figure S1). The field was prepared for sowing (ploughing and disking) each year after oilseed rape cultivation, and seeds were sown on 28 March 2020, 11 April 2021, and 24 March 2022. Neither growth regulators nor fungicides were applied during the growing season. Throughout the cultivation seasons, we did not observe infectious pressure from pathogens. Fertilizer (KCl) equivalent to a potassium dose of 0, 100, and 200 kg/ha, respectively, was applied to marked plots as the only fertilizer. Once the plants started to emerge, the *S. indica* inoculum was homogeneously distributed across the selected plots. Soil moisture was measured with TMS4 probes (Tomst, Prague, Czech Republic), applied at depths of 20 and 40 cm below the soil surface (Figure 1). The macronutrient content of the unfertilized soil was analyzed according to the Mehlich III methodology (Table S1).

### 2.2. Inoculum Preparation and Colonization Evaluation

**Figure 2:**
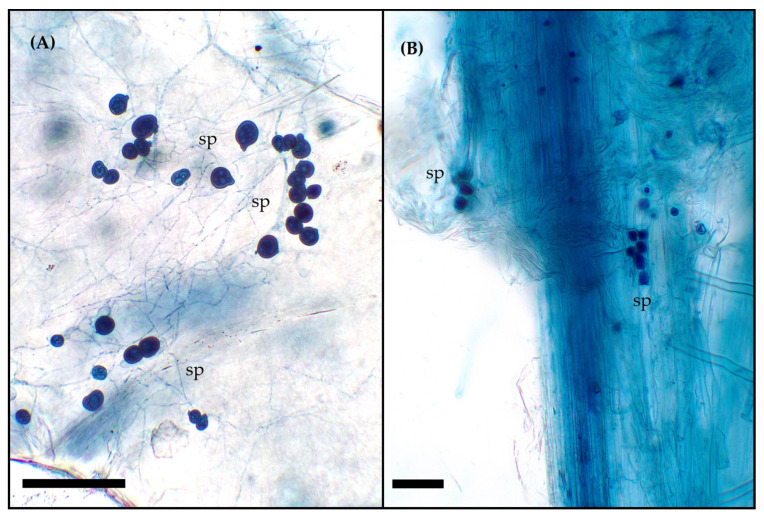
(**A**) *Serendipita indica* mycelium and spores (sp.) on vermiculite particle used as inoculum; (**B**) spores (sp.) of *S. indica* in spring barley roots. Stained with trypan blue, the bar represents 50 μm.

The process of creating the inoculum for *S. indica* fungus was initiated by saturating vermiculite with 1/3 liquid broth medium solution (Carl Roth GmbH + Co. KG, Karlsruhe, Germany), adding of 7 g/L of sucrose, sterilizing in plastic bags, and adding the culture of *S. indica* fungus (CBS culture collection—CBS 125645, Utrecht, The Netherlands). The fungus was cultured in the substrate for two months at 20 °C in the dark, after which it had grown throughout the content of each bag and formed spores (Figure 2A). This inoculum was applied to emerging plants in the field at a rate of 200 g of inoculum per square meter. In the milky ripeness stage, roots were taken for microscopic evaluation of colonization by the endophytic fungus. The roots were fixed in 70% ethanol, cleared in 2.5% KOH, acidified with 1% HCl, and stained with 0.05% trypan blue in lactoglycerol. The percentage of root colonization was determined at 100x magnification, visible spores were evaluated (Figure 2B), and the identity of the fungus was confirmed using PCR products using species-specific primers (Figure S2) according to Bütehorn et al. (2000) [45]. The PCR reaction was carried out in 20 μL volumes containing 10 ng of total DNA from spring barley roots. The reaction mixture contained 0.2 mM of dNTP, 0.2 mM, 1 U of Taq polymerase (Thermo Fisher Scientific, Waltham, MA, USA), and primers. Reaction buffer consisted of 75 mM Tris-HCl, 20 mM (NH_4_)_2_SO_4_, and 2.5 mM MgCl_2_ (Thermo Fisher Scientific, Waltham, MA, USA). PCR was carried out under the following cycling conditions: initial denaturation at 94 °C for 6 min, 35 cycles of denaturation (94 °C, 30 s), annealing (58 °C, 30 s), extension (72 °C, 45 s), and the final extension at 72 °C for 1 min. The PCR products (10 μL) were analyzed using agarose gel electrophoresis.

### 2.3. Analyzed Parameters

The first sampling of plants for sugars content was carried out in the early tillering stage in BBCH 21–23 [46], and the second sampling 2–3 weeks later in the early stem elongation stage (BBCH 31–34)—in this text, these are considered as soluble sugars I and soluble sugars II, respectively (Figure 1). Three plants were taken from each plot and a mixed sample was prepared. The samples were dried and analyzed by the colorimetric method for the determination of sugars using a phenol-sulfuric acid reaction according to Dubois et al. (1956) [47] and expressed as milligrams per gram of dry weight. The difference in soluble sugars content between sampling I and II was evaluated as Δ soluble sugars. To determine the stable carbon isotope ratio δ^13^C in plant tissue, a mixed sample of ten flag leaves per plot was prepared in the milky ripeness stage. The samples were dried and pulverized, and the isotopic ratio was analyzed at UC Davis, California, Stable Isotope Facility, on a mass spectrometer (Sercon Ltd., Cheshire, UK). The length and width of the second upper leaf were measured on ten randomly selected plants from each plot and the average of these measurements was calculated per plot. The heights of the plants were measured in a bundle of multiple tillers from several plants, three times per plot. The number of tillers per square meter was calculated from three repeated measurements of the number of tillers per meter length of uniform vegetation. The experiment was harvested at full maturity on 1 August 2020, 8 August 2021, and 26 July 2022, respectively, the yield per plot (kg) was evaluated, the weight of thousand grains—TGW (g)—was measured, and the starch content of the grains (%) was analyzed according to Ewers’ polarimetric method (ISO 10520; [48]).

### 2.4. Statistical Analysis

Statistical analyses were performed in STATISTICA 13 (TIBCO Software Inc., Palo Alto, CA, USA). The data obtained were subjected to factorial analysis of variance (ANOVA). Due to the low number of replicates, the factorial ANOVA was performed in the three-degree interaction scheme. The Tukey post-hoc test was used to obtain statistical significances. Prior to ANOVA, the homogeneity of variances was tested by combined Bartlett’s, Cochran’s, and Hartley’s tests, and the normality of the residuals was tested using the Shapiro–Wilk test. The data were transformed by logarithmic or BoxCox transformation, if needed. Productivity parameters such as yield per plot (kg), TGW (g), and starch in grains (%); growth parameters such as tillers/m^2^, leaf length (cm), leaf width (mm), and plant height (cm); and physiological parameters such as soluble sugars I, soluble sugars II, Δ soluble sugars, and δ^13^C were evaluated. Data are presented as arithmetic mean ± standard deviation (*SD*).

## 3. Results

The production parameters are of primary interest in spring barley cultivation. Growth and physiological parameters are also an essential part of the evaluation of barley genotypes’ adaptations to environmental conditions. The statistical significances of these parameters in relation to experimental factors and their interactions are presented in Table 1.

**Figure 3:**
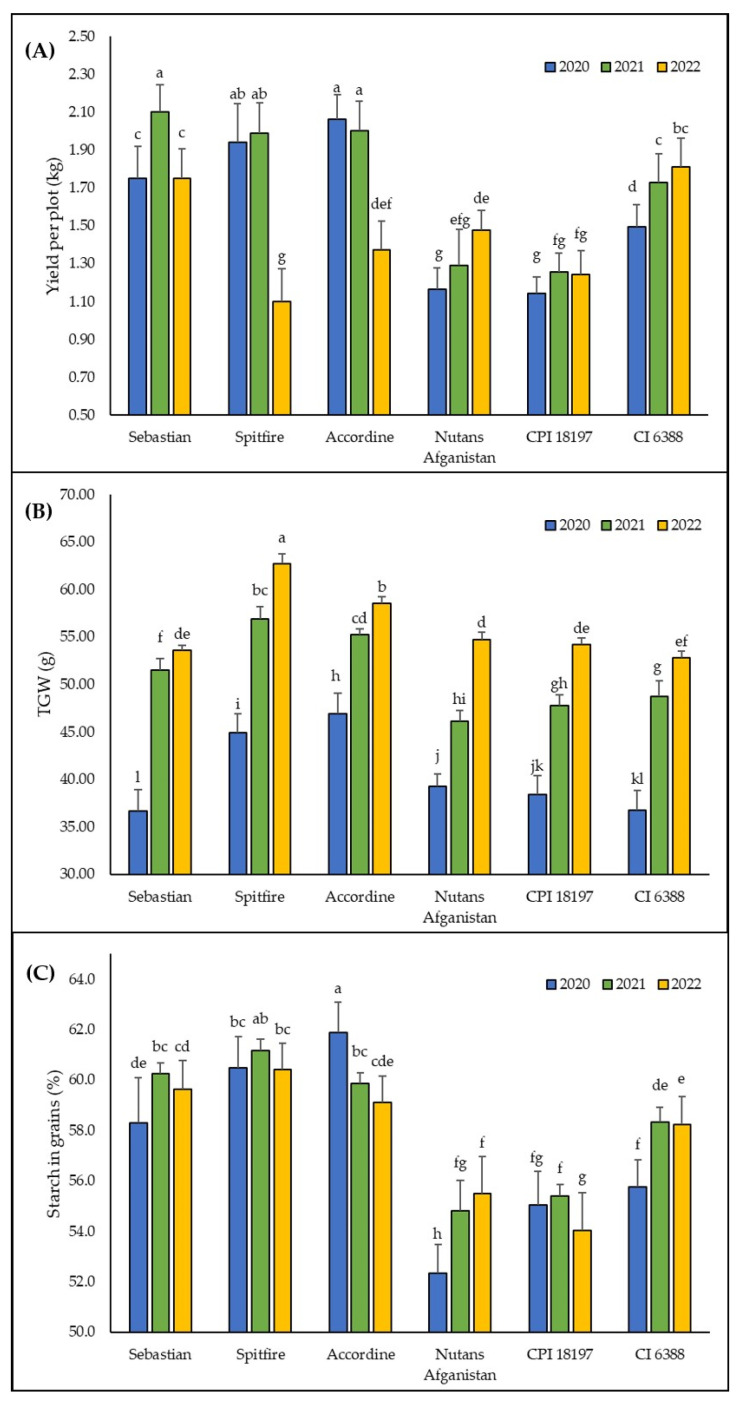
Productivity parameters of genotypes in different seasons. (**A**) yield per plot, (**B**) thousand grains weight (TGW), and (**C**) content of starch in grains. Columns represent means, bars *SD* (*n* = 15) followed by the same letter if there was no statistical difference according to the Tukey_0.05_ test.

**Figure 4:**
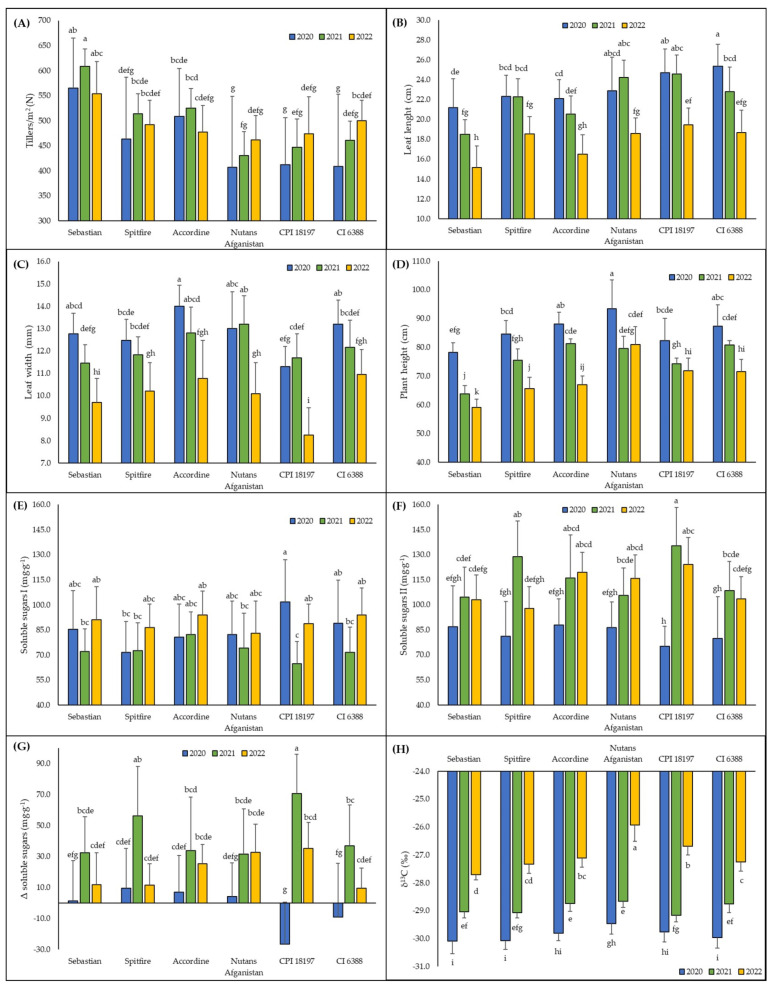
Growth and physiological parameters among genotypes of spring barley during cultivation seasons. (**A**) tillers/m^2^, (**B**) leaf length, (**C**) leaf width, (**D**) plant height, (**E**) soluble sugars I, (**F**) soluble sugars II, (**G**) Δ soluble sugars, and (**H**) δ^13^C. Columns represent means, bars *SD* (*n* = 15) followed by the same letter if there was no statistical difference according to the Tukey_0.05_ test.

The results of three seasonal experiments demonstrate that the yield of the spring barley, as well as TGW, starch content in grains, tillering, length and width of leaves, plant height, and some physiological parameters, namely, the soluble sugars II content and carbon isotope signature, differed among different genotypes used (Table 2A). The different seasonal environmental conditions had statistically significant effects on all measured parameters (Table 1 and Table 2B). The effects of interaction between genotype and season factors were both statistically significant for all productivity parameters (Figure 3), as well as for all growth and physiological parameters (Figure 4). The effect of this interaction on the yield per plot was observed; even when considering the seasons as a random factor (*F* = 84.080; *p* < 0.001), this interaction contributed to the overall variability of the yield data by 72%.

There is a statistically significant effect of fertilization on yield found in 2022 when the higher dose of potassium fertilizer decreases the yield (1.40 ± 0.30 kg/plot) compared to unfertilized treatments (1.53 ± 0.28 kg/plot). No similar effect is observed in the 2020 and 2021 seasons (Figure S3A). There is a similar negative effect of potassium fertilization on TGW, but only in the 2020 season and for *S. indica*-inoculated plants (39.27 ± 3.81 g for 200 kg/ha, 41.98 ± 3.86 g for unfertilized plants; Figures S3B and S4A, Table S2). However, compared to the effect of cultivation season (Table 2B) or the effect of genotype × season interaction (Figure 3B), this variation in TGW is negligible. The same pattern is found for the content of starch in grains, for which there is a statistically significant interaction of fertilization and season factors (Table 1A), but the effect is weak (Figure S3C) compared to the effect of genotype × season interaction (Figure 3C).

Fertilization with both potassium rates results in a statistically significant reduction of the number of tillers per square meter (468 ± 84 for 100 kg/ha, 465 ± 106 for 200 kg/ha) compared to unfertilized plants (518 ± 82). This effect is, however, observed only for the 2020 season, while in 2021 and 2022, there is no effect of potassium fertilization on tillering (Figure S3D). This effect is however further modified by genotype identity (Table S4, Figure S5A). Tillering in response to fertilization is significantly affected also by genotype identity (Figure S6A) and *S. indica* inoculation (Table S3, Figure S7).

The leaves of unfertilized plants are statistically significantly wider (12.0 ± 1.8 mm) than those of plants fertilized with both potassium rates (11.4 ± 1.8 mm for 100 kg/ha, 11.5 ± 1.9 mm for 200 kg/ha). The effect of fertilization, together with the effect of genotype identity, has a statistically significant effect on plant height under different seasonal conditions (Table S4, Figure S5B). Plant height in response to fertilization is also affected by *S. indica* inoculation under different seasonal conditions (Table S2, Figure S4B). Inoculation with endophytic fungus itself does not have any significant effect on yield or growth parameters. However, it is found to have a significant effect on the soluble sugars I parameter, reducing its content in plant tissues (79.2 ± 18.8 mg·g^−1^) compared to plants without inoculation (84.7 ± 20.8 mg·g^−1^). In the difference between sampling I and sampling II (Δ soluble sugars), the effect of inoculation is observed to increase soluble sugars levels in plant tissues (25.1 ± 32.5 mg·g^−1^) compared to those of plants without endophytic association (18.0 ± 32.6 mg·g^−1^). The leaf length of barley genotypes is affected by presence of *S. indica* in their roots (Figure S6B). Colonization of spring barley roots by *S. indica* is found to be between 10–15% of root length in all seasons.

The isotope signature (δ^13^C) is statistically significantly affected by fertilization and genotype identity during all seasons (Table S4, Figure S5C).

## 4. Discussion

The evaluation of plant stress under natural conditions is a complex task that requires multiple approaches due to the high variability of environmental factors, such as irradiance, temperature, and soil conditions, which can temporarily act as strong stressors. Additionally, other stressors, including biotic and abiotic factors, and plant developmental stages can further complicate stress assessment. Moreover, most methodologies fail to capture the combined effects of stressors and the exact plant response to them, and the temporal variability of plant responses limits the use of molecular methods for stress detection. Although spectral reflectance indices are often used for non-destructive stress assessment, they also have limitations [49].

These challenges were faced in this study, which aimed to evaluate six highly contrasting spring barley genotypes under realistic field conditions. Drought-stress tolerance characteristics were not available for all genotypes under investigation. The responses of genotypes to drought stress exhibit variability. For instance, the cultivar Sebastian is acknowledged for its drought tolerance [50,51,52]. However, when compared to other cultivars, its responsiveness is limited to specific physiological parameters, displaying an average reaction to drought stress [53].

The year 2020 was characterized by a remarkably high amount of precipitation, registering 125% of the average rainfall for the previous 30 years from January to July. Specifically, the cultivation period experienced an extremely wet May, with a rainfall of 158%, and a highly saturated June, with a rainfall of 187% in comparison to the 30-year monthly average rainfall. The impact of precipitation on yield was significant, especially for genotypes originating from arid and warm regions, where root waterlogging and lower average temperature might have influenced yield. The newer variety Sebastian also exhibited a reduction in yield in response to the wet conditions.

In contrast, the 2021 cultivation season was poorer in precipitation during plant growth than 2020, but was considered normal in this study and corresponded to the highest yields of spring barley. From January to July, rainfall was 76% of the 30-year average rainfall, with wet January and February and normal April and May, followed by dry periods in June and July. Despite this, there was sufficient moisture available for the plants during the early stages of development.

The first three months of 2022 experienced lower-than-normal rainfall, with a significant drought occurring in May, i.e., during the critical developmental stages of barley in an otherwise normal rainfall season. Spike primordia differentiate from the early heading stage [54,55]. This is a critical part in plant development and reproductive processes, influencing future yield potential [56]. Stress conditions can lead to a reduction in the cell division rate and degradation of spike primordia, caused by an increase in abscisic acid concentration in the apical and basal parts of the spikes. This physiological response can result in the shortened duration of developmental stages and lead to a decrease in seed count [57]. The reduction in grain formation was indicated by a decrease in yield, but later conditions enabled an increase in TGW (Figure 3B). Overall precipitation from January to the end of the cultivation period in July was only 65% of the 30-year average.

Rainfall averages and sums are not a decisive determinant of crop growth and production; more important is the actual distribution of available moisture during the growing season, especially at critical developmental stages. Contributing factors may be the saturation of deeper soil profiles from previous seasons or, conversely, light rainfall not detected by probes in the measured soil depths, high temperature, evaporation rates, and others. The 2021 and 2022 seasons were very similar in terms of overall rainfall, but we observed significantly different results in yields and other productivity parameters. The good yield results of the 2021 season may have been caused by the availability of sufficient rainfall after short periods of drought, which is evident from peaks at 20 cm depth, despite no moisture seeping into the lower soil profile (Figure 1B). Because of the different water requirements at different growth stages and the regenerative capacity of plants, the isotope signature can be considered the most appropriate indicator of the effect of drought on plants.

The significantly contrasting climatic conditions experienced during the three contrasting growing seasons were clearly evident in the considerable year-to-year variability observed in yields. Among the genotypes evaluated, the most stable, high yielding variety was Sebastian. Its yield remained almost identical in the wet year 2020 and the dry year 2022 with values 1.75 ± 0.17 kg/plot in 2020, and 1.75 ± 0.16 kg/plot in 2022, respectively, and was 83% of its yield in 2021, which was the highest yield recorded in these experiments. The other two modern varieties, Spitfire and Accordine, demonstrated notable yield reductions in the drier conditions of 2022, reaching only 55% and 69%, respectively, of their yields in 2021. On the contrary, genetic sources produced less yields, but were not influenced by drought conditions. It can be assumed that genetic resources from arid regions that are not bred for yield maximization will have lower production parameters; despite this, CI 6388 appears to be a very valuable resource in regard to yield (Table 2A).

Although episodes of water shortage can be considered the dominant stressor, especially in the 2022 season (as shown by the carbon isotope signature values and, partly, by differences in the content of soluble sugars between growing seasons, in addition to the soil water content itself), the plants were undoubtedly also exposed to other stressors during growth, such as high temperature or irradiance. All these factors contributed to the performance of the plants. To assess the actual effects of stressors, it is preferable to sample different stages of plant growth. Therefore, we conducted two samplings for the analysis of soluble sugars in the early stage of plant growth to evaluate the current functioning of the assimilation apparatus.

The soil conditions during the sampling of plants for soluble sugars analysis varied across each year, reflecting the influence of factors such as stress history, soil preparation, and temperature. In 2020, the first sampling occurred under conditions of adequate soil moisture, while the second sampling took place under even higher moisture levels. These values can be considered as a baseline level of soluble sugars in the tissues of the cultivars that were not subjected to drought stress. The decrease observed in the Δ soluble sugars parameter in the genotype CPI 18197 may be attributed to metabolic adjustments in this genotype prior to the first sampling in response to environmental factors. In the 2021 season, the first sampling was conducted shortly after a period of peak moisture, indicating that the plants were likely not yet experiencing significant stress. Although the second sampling also followed rainfall, the soil moisture profile was considerably lower, resulting in a substantial increase in the Δ soluble sugars parameter. During the 2022 season, sampling occurred during a period without notable fluctuations in soil moisture. The values of Δ soluble sugars observed reflected the cumulative effects of prolonged exposure to stressors on the physiological condition of the spring barley genotypes. The variability in soluble sugars levels and the patterns observed in the Δ soluble sugars parameter provided insights into the physiological responses of the genotypes to the varying stress conditions experienced over the season.

In addition to collecting samples for soluble sugars analysis, we also obtained samples for stable carbon isotope signature analysis at the milky maturity stage of the grains. These analyses offer valuable insights into the performance of genotypes under stressor exposure. By examining the stable carbon isotope signatures, we gain crucial information about the physiological responses and carbon assimilation efficiency of the genotypes during periods of stress. This analysis provides a deeper understanding of the adaptive mechanisms employed by the genotypes and their ability to cope with challenging environmental conditions.

In our study, we aimed to evaluate the response of plants to real field conditions using integral variables, rather than focusing on the effects of specific stressors. Our primary interest was in the yield, and we found significant inter-genotype differences in this parameter. Although the genetic resources we used were not as productive as modern varieties bred primarily for yield, their traits suggest that they could be a promising source of genes for breeding, particularly to increase tolerance to abiotic stresses, as suggested by the 2022 season. The response of spring barley to environmental conditions is a genotype-specific trait, so there are considerable differences for many parameters [58]. Our results also demonstrate the strong effects of genotypes compared to the effects of fertilizer and endophyte applications on the measured parameters.

This study aimed to evaluate the yield potential of the selected genotypes under real climatic conditions without their manipulation, and since the previous few years were characterized by very low rainfall and high temperatures, we expected that drought would be a very strong stressor. To ameliorate the effects of water stress, potassium fertilizers can be used, as they serve as an osmoticum for plants and can, therefore, influence or regulate their water status [31]. Agronomic methods to increase crop drought resilience include biopriming and osmopriming. These methods involve allowing the grain to swell for some time in a specific environment with a symbiotic organism or with an osmotically active substance such as potassium. The priming of seeds induces metabolic processes that enable faster and better germination in plants under stressful conditions [59,60,61]. Fertilizer application before sowing can act as a form of osmopriming and promote grain filling at later stages of growth.

The experimental factors of fertilization and inoculation were evaluated, and further investigation is necessary to fully understand their effects on individual genotypes. Among the primary parameters measured, yield was not significantly influenced by the application of K fertilizer, except in the year 2022, while tillering and leaf width were affected. Therefore, it cannot be assumed that increasing K fertilizer rates will consistently promote drought tolerance, as this must be assessed on a case-by-case basis, considering the specific circumstances and cost–benefit analysis. Our findings also indicate that the interaction between potassium content and seasonal changes influenced tillering in barley plants. However, it is important to note that various other factors, including environmental conditions, should be considered when interpreting these results. Future research should further investigate the specific environmental conditions influencing the response of spring barley genotypes to drought stress. This may involve examining factors like temperature fluctuations or soil properties to deepen our understanding of genotype–environment interactions and stress resilience. Furthermore, future studies should investigate alternative stress resilience strategies, including the utilization of other beneficial microorganisms or sustainable nutritional approaches. The endophytic fungus *S. indica*, which is indigenous to the arid regions of India, may have the ability to alter plant responses to water scarcity and high temperatures. We observed its effect on Δ soluble sugars, where inoculated plants showed a higher increase in soluble sugars between sampling I and II than non-inoculated plants. Thus, a positive effect on drought tolerance metabolic processes can be hypothesized. However, the content of soluble sugars I in colonized plants decreased, which can possibly be explained by the transfer of metabolites from the plant to the fungus. The formation of a soil environment with symbiotic microorganisms has an impact on soil quality and function because drought stress is not only associated with lack of rainfall, but also with the water retention capacity of soil and soil microorganisms [20]. In our region, *S. indica* is not a native species; therefore, its successful introduction to experimental sites and the formation of an endophytic association may also be influenced by local conditions. Nonetheless, in all the years in which the experiment was carried out, barley plants were effectively inoculated with the endophyte, and no differences were observed between seasons and treatments. The potential of endophyte inoculation compared to arbuscular fungi remains to be adequately studied, and extensive experimental research is necessary.

While the current study focused on spring barley genotypes, extending these findings to other crop species would enhance our understanding of stress tolerance mechanisms. Employing similar methodologies and interventions across different crops enables researchers to assess the transferability of strategies for improving stress resilience in a broader agricultural context.

## 5. Conclusions

This study aims to provide insight into potential options for stabilizing crop production under non-standard climatic conditions through the selection of suitable plant material and the use of agronomic interventions. In addition, the article explored the physiological responses of spring barley plants to varying conditions throughout the cultivation period, with the goal of identifying potential breeding sources for genetically determined tolerance to abiotic stress. The study found that the genotype and year of cultivation were the principal factors affecting the selected parameters, highlighting the importance of selecting the right plant material for production under non-standard climatic conditions. The plants’ response to environmental conditions was significant, with modern genotypes experiencing reduced yields under conditions of insufficient moisture, as opposite to the cultivars from arid regions, which were either unaffected or even had a higher yield. Fertilization and inoculation were found to partially contribute to the dominant factors of genotype and season. However, the effectiveness of these interventions may require different conditions to enhance their influence. Overall, this research highlights the importance of selecting appropriate plant material and employing tailored agronomic strategies to stabilize crop production under diverse climatic conditions, offering insights into potential genetic sources for abiotic stress tolerance and contributing to sustainable barley cultivation practices.

## Figures and Tables

**Table 1:** (**A**–**C**): Results of factorial ANOVAs evaluating the effects of six spring barley genotypes, application of potassium fertilizer, inoculation with *Serendipita indica*, and three cultivation seasons on the productivity, growth, and physiological parameters. The *F* and *p* values are presented, and statistically significant effects (*p* < 0.05) are marked with an asterisk. TGW—weight of thousand grains; δ^13^C—carbon isotope signature.

(A)		Yield per Plot (kg)		TGW (g)		Starch in Grains (%)			
Factor		*F*	*p*	*F*	*p*	*F*	*p*		
Genotype (GE)		141.806	<0.001 *	358.560	<0.001 *	288.541	<0.001 *		
Fertilization (FE)		0.236	0.790	2.148	0.120	2.803	0.063		
Inoculation (IN)		0.091	0.763	0.465	0.496	0.564	0.454		
Season (SE)		72.215	<0.001 *	3022.513	<0.001 *	19.764	<0.001 *		
GE × FE		0.515	0.878	0.871	0.562	1.652	0.095		
GE × IN		1.064	0.382	1.078	0.374	1.774	0.120		
FE × IN		0.122	0.885	1.385	0.253	0.030	0.970		
GE × SE		49.682	<0.001 *	25.628	<0.001 *	18.164	<0.001 *		
FE × SE		4.465	0.002 *	3.428	0.010 *	2.876	0.024 *		
IN × SE		0.294	0.746	0.189	0.828	0.690	0.503		
GE × FE × IN		0.738	0.688	0.663	0.758	0.597	0.815		
GE × FE × SE		0.838	0.665	1.378	0.138	1.235	0.231		
GE × IN × SE		0.746	0.680	0.956	0.483	0.906	0.529		
FE × IN × SE		0.390	0.816	3.686	0.007 *	0.792	0.532		
**(B)**		**Tillers/m^2^** **(N)**		**Leaf Length** **(cm)**		**Leaf Width** **(mm)**		**Plant Height** **(cm)**	
**Factor**		***F***	***p***	***F***	***p***	***F***	***p***	***F***	***p***
Genotype (GE)		31.389	<0.001 *	31.215	<0.001 *	19.699	<0.001 *	81.148	<0.001 *
Fertilization (FE)		21.168	<0.001 *	0.959	0.385	7.113	0.001 *	2.521	0.083
Inoculation (IN)		0.729	0.394	0.853	0.357	0.180	0.672	0.120	0.729
Season (SE)		9.395	<0.001 *	169.498	<0.001 *	139.924	<0.001 *	287.943	<0.001 *
GE × FE		2.215	0.019 *	1.874	0.051	0.991	0.453	0.780	0.648
GE × IN		1.279	0.275	2.634	0.025 *	1.355	0.244	0.730	0.602
FE × IN		0.214	0.807	2.653	0.073	1.369	0.257	0.182	0.834
GE × SE		2.305	0.014 *	2.447	0.009 *	2.709	0.004 *	9.021	<0.001 *
FE × SE		20.911	<0.001 *	1.995	0.097	1.614	0.173	1.545	0.191
IN × SE		0.203	0.817	1.798	0.169	1.037	0.357	0.299	0.742
GE × FE × IN		2.756	0.003 *	1.609	0.107	1.019	0.429	0.287	0.984
GE × FE × SE		2.030	0.008 *	1.113	0.339	0.853	0.646	2.272	0.002 *
GE × IN × SE		1.525	0.134	1.404	0.182	0.721	0.704	0.963	0.477
FE × IN × SE		0.380	0.823	0.610	0.656	0.745	0.563	2.967	0.021 *
**(C)**		**Soluble Sugars I (mg·g^−1^)**		**Soluble Sugars II (mg·g^−1^)**		**Δ soluble Sugars (mg·g^−1^)**		**δ^13^C** **(‰)**	
**Factor**		***F***	***p***	***F***	***p***	***F***	***p***	***F***	***p***
Genotype (GE)		1.763	0.123	3.386	0.006 *	2.154	0.061	40.423	<0.001 *
Fertilization (FE)		2.624	0.075	2.753	0.066	0.680	0.508	0.050	0.951
Inoculation (IN)		5.615	0.019 *	0.489	0.485	5.244	0.023 *	0.779	0.379
Season (SE)		18.223	<0.001 *	82.481	<0.001 *	74.047	<0.001 *	1673.900	<0.001 *
GE × FE		1.321	0.222	0.488	0.896	1.342	0.211	0.852	0.579
GE × IN		0.660	0.654	0.417	0.836	0.230	0.949	0.940	0.456
FE × IN		0.244	0.784	0.219	0.803	0.476	0.622	0.085	0.918
GE × SE		2.282	0.015 *	4.691	<0.001 *	5.519	<0.001 *	11.492	<0.001 *
FE × SE		1.267	0.285	1.388	0.240	1.338	0.258	0.511	0.728
IN × SE		0.030	0.970	0.542	0.582	0.254	0.776	0.180	0.835
GE × FE × IN		0.547	0.855	1.483	0.149	0.488	0.897	1.683	0.088
GE × FE × SE		0.764	0.754	0.865	0.631	0.967	0.505	1.657	0.044 *
GE × IN × SE		0.393	0.949	0.766	0.661	0.597	0.815	0.334	0.971
FE × IN × SE		0.622	0.648	0.066	0.992	0.190	0.943	0.564	0.689

**Table 2:** Evaluation table of measured parameters for the genotype factor (**A**) and for the season factor (**B**). Data represent means ± *SD* (*n* = 15), columns marked by the same letter are not statistically different according to the Tukey_0.05_ test.

(A)	Genotype	Yield Per Plot (kg)	TGW (g)	Starch in Grains (%)	Tillers/m^2^ (N)	Leaf Length (cm)	Leaf Width (mm)	Plant Height (cm)	Soluble Sugars I (mg·g^−1^)	Soluble Sugars II (mg·g^−1^)	Δ Soluble Sugars (mg·g^−1^)	δ^13^C (‰)
Sebastian		1.87 ± 0.23 ^a^	47.24 ± 7.77 ^c^	59.4 ± 1.5 ^b^	576 ± 74 ^a^	18.3 ± 3.3 ^d^	11.3 ± 1.6 ^c^	67.0 ± 8.7 ^e^	82.8 ± 20.4 ^a^	98.1 ± 20.8 ^b^	15.3 ± 26.2 ^a^	−28.9 ± 1.0 ^d^
Spitfire		1.68 ± 0.45 ^b^	54.85 ± 7.64 ^a^	60.7 ± 1.0 ^a^	490 ± 81 ^bc^	21.1 ± 2.6 ^b^	11.5 ± 1.4 ^bc^	75.2 ± 8.9 ^d^	76.8 ± 17.6 ^a^	102.6 ± 27.1 ^ab^	25.8 ± 32.7 ^a^	−28.8 ± 1.2 ^cd^
Accordine		1.81 ± 0.35 ^a^	53.52 ± 5.12 ^b^	60.3 ± 1.5 ^a^	504 ± 68 ^b^	19.7 ± 3.0 ^c^	12.5 ± 1.9 ^a^	78.8 ± 9.4 ^bc^	85.6 ± 16.8 ^a^	107.7 ± 23.3 ^ab^	22.1 ± 27.1 ^a^	−28.5 ± 1.2 ^b^
Nutans Afganistan		1.31 ± 0.19 ^c^	46.71 ± 6.48 ^cd^	54.2 ± 1.9 ^d^	433 ± 91 ^d^	21.9 ± 3.4 ^ab^	12.1 ± 2.0 ^ab^	84.7 ± 9.5 ^a^	79.8 ± 20.0 ^a^	102.6 ± 19.4 ^ab^	22.8 ± 26.4 ^a^	−28.0 ± 1.6 ^a^
CPI 18197		1.21 ± 0.12 ^d^	46.77 ± 6.68 ^cd^	54.8 ± 1.3 ^d^	444 ± 79 ^d^	22.9 ± 3.2 ^a^	10.4 ± 1.9 ^d^	76.1 ± 6.9 ^cd^	85.1 ± 23.3 ^a^	111.5 ± 31.5 ^a^	26.4 ± 46.7 ^a^	−28.5 ± 1.4 ^b^
CI 6388		1.68 ± 0.19 ^b^	46.08 ± 7.03 ^d^	57.4 ± 1.5 ^c^	457 ± 95 ^cd^	22.3 ± 3.6 ^a^	12.1 ± 1.4 ^ab^	79.9 ± 8.2 ^b^	84.8 ± 21.5 ^a^	97.3 ± 22.6 ^b^	12.5 ± 31.8 ^a^	−28.7 ± 1.2 ^bc^
**(B)**	**Season**	**Yield Per Plot (kg)**	**TGW** **(g)**	**Starch in Grains (%)**	**Tillers/m^2^** **(N)**	**Leaf Length (cm)**	**Leaf Width (mm)**	**Plant Height (cm)**	**Soluble Sugars I (mg·g^−1^)**	**Soluble Sugars II (mg·g^−1^)**	**Δ Soluble Sugars (mg·g^−1^)**	**δ^13^C** **(‰)**
2020		1.59 ± 0.38 ^b^	40.48 ± 4.43 ^c^	57.3 ± 3.5 ^c^	461 ± 129 ^b^	23.1 ± 2.9 ^a^	12.8 ± 1.4 ^a^	85.6 ± 8.1 ^a^	85.1 ± 23.5 ^a^	82.9 ± 19.5 ^b^	−2.2 ± 28.9 ^c^	−29.9 ± 0.4 ^c^
2021		1.73 ± 0.37 ^a^	51.04 ± 4.11 ^b^	58.3 ± 2.5 ^a^	497 ± 74 ^a^	22.2 ± 2.8 ^b^	12.2 ± 1.2 ^b^	75.9 ± 6.7 ^b^	72.9 ± 16.2 ^b^	116.4 ± 23.3 ^a^	43.5 ± 31.5 ^a^	−28.9 ± 0.3 ^b^
2022		1.46 ± 0.29 ^c^	56.07 ± 3.59 ^a^	57.8 ± 2.6 ^b^	493 ± 62 ^a^	17.8 ± 2.4 ^c^	10.0 ± 1 0.6 ^c^	69.3 ± 7.9 ^c^	89.5 ± 16.1 ^a^	110.6 ± 16.7 ^a^	21.1 ± 18.8 ^b^	−27.0 ± 0.7 ^a^

## Data Availability

All data are contained within the article and Supplementary Materials.

## Supplementary Materials

The following supporting information can be downloaded at https://www.mdpi.com/article/10.3390/plants13081168/s1: Figure S1—mean temperatures and precipitation in growing seasons; Table S1—the nutrient content in the soil taken at the experimental site before experiments; Figure S2—electrophoresis gel of the PCR products using *Serendipita indica*-specific primers; Figure S3—interaction of fertilization and season on productive parameters and tillering; Table S2—interaction of fertilization, inoculation, and season factors on the thousand grains weight and plant height; Figure S4—interaction of fertilization, inoculation, and season factors on the thousand grains weight (TGW) and plant height; Table S3—interaction of genotype, fertilization, and season factors on the tillering, plant height, and carbon isotope signature (δ^13^C); Figure S5—interaction of genotype, fertilization, and season factors on the tillering, plant height, and carbon isotope signature (δ^13^C); Figure S6—tillering of different genotypes in interaction with fertilization and leaf length in interaction with genotype and inoculation; Table S4—interaction of genotype, fertilization, and inoculation factors on the tillering; Figure S7—interaction of genotype, fertilization, and inoculation factors on the tillering.

## References

[1] Shakoor U., Saboor A., Ali I., Mohsin A.Q. (2011). Impact of Climate Change on Agriculture: Empirical Evidence from Arid Region. Pak. J. Agric. Sci..

[2] Dodd I.C., Ryan A.C. (2016). Whole-Plant Physiological Responses to Water-Deficit Stress. eLS—Encyclopedia of Life Sciences.

[3] Osakabe Y., Osakabe K., Shinozaki K., Tran L.-S.P. (2014). Response of Plants to Water Stress. Front. Plant Sci..

[4] Hermans K., McLeman R. (2021). Climate Change, Drought, Land Degradation and Migration: Exploring the Linkages. Curr. Opin. Environ. Sustain..

[5] Kaur H., Kohli S.K., Khanna K., Bhardwaj R. (2021). Scrutinizing the Impact of Water Deficit in Plants: Transcriptional Regulation, Signaling, Photosynthetic Efficacy, and Management. Physiol. Plant.

[6] Trnka M., Semerádová D., Novotný I., Dumbrovský M., Drbal K., Pavlík F., Vopravil J., Štěpánková P., Vizina A., Balek J. (2016). Assessing the Combined Hazards of Drought, Soil Erosion and Local Flooding on Agricultural Land: A Czech Case Study. Clim. Res..

[7] Kibblewhite M.G., Ritz K., Swift M.J. (2008). Soil Health in Agricultural Systems. Phil. Trans. R. Soc. B.

[8] Wyka T.P., Bagniewska-Zadworna A., Kuczyńska A., Mikołajczak K., Ogrodowicz P., Żytkowiak M., Surma M., Adamski T. (2019). Drought-Induced Anatomical Modifications of Barley (*Hordeum vulgare* L.) Leaves: An Allometric Perspective. Environ. Exp. Bot..

[9] Zulfiqar F., Younis A., Riaz A., Mansoor F., Hameed M.A., Akram N.A., Abideen Z. (2020). Morpho-anatomical adaptations of two *Tagetes erecta* L. cultivars with contrasting response to drought stress. Pak. J. Bot..

[10] Basu S., Ramegowda V., Kumar A., Pereira A. (2016). Plant Adaptation to Drought Stress. F1000Research.

[11] Chaves M.M., Maroco J.P., Pereira J.S. (2003). Understanding Plant Responses to Drought—From Genes to the Whole Plant. Funct. Plant Biol..

[12] Shankar Naik B. (2019). Functional Roles of Fungal Endophytes in Host Fitness during Stress Conditions. Symbiosis.

[13] Singh L.P., Gill S.S., Tuteja N. (2011). Unraveling the Role of Fungal Symbionts in Plant Abiotic Stress Tolerance. Plant Signal. Behav..

[14] Ghaffari M.R., Mirzaei M., Ghabooli M., Khatabi B., Wu Y., Zabet-Moghaddam M., Mohammadi-Nejad G., Haynes P.A., Hajirezaei M.R., Sepehri M. (2019). Endophytic Fungus Piriformospora Indica Improves Drought Stress Adaptation in Barley by Metabolic and Proteomic Reprogramming. Environ. Exp. Bot..

[15] Murphy B.R., Martin Nieto L., Doohan F.M., Hodkinson T.R. (2015). Fungal Endophytes Enhance Agronomically Important Traits in Severely Drought-stressed Barley. J. Agron. Crop Sci..

[16] Bleša D., Matušinský P., Sedmíková R., Baláž M. (2021). The Potential of Rhizoctonia-Like Fungi for the Biological Protection of Cereals against Fungal Pathogens. Plants.

[17] Zhang Y., Yu X., Zhang W., Lang D., Zhang X., Cui G., Zhang X. (2019). Interactions between Endophytes and Plants: Beneficial Effect of Endophytes to Ameliorate Biotic and Abiotic Stresses in Plants. J. Plant Biol..

[18] Baron N.C., Rigobelo E.C. (2022). Endophytic Fungi: A Tool for Plant Growth Promotion and Sustainable Agriculture. Mycology.

[19] Hong Y., Zhang G. (2020). The Influence of Drought Stress on Malt Quality Traits of the Wild and Cultivated Barleys. J. Integr. Agric..

[20] Tyagi J., Chaudhary P., Mishra A., Khatwani M., Dey S., Varma A. (2022). Role of Endophytes in Abiotic Stress Tolerance: With Special Emphasis on *Serendipita indica*. Int. J. Environ. Res..

[21] Ghaffari M.R., Ghabooli M., Khatabi B., Hajirezaei M.R., Schweizer P., Salekdeh G.H. (2016). Metabolic and Transcriptional Response of Central Metabolism Affected by Root Endophytic Fungus *Piriformospora indica* under Salinity in Barley. Plant Mol. Biol..

[22] Achatz B., Kogel K.-H., Franken P., Waller F. (2010). *Piriformospora indica* Mycorrhization Increases Grain Yield by Accelerating Early Development of Barley Plants. Plant Signal. Behav..

[23] Bago B., Pfeffer P.E., Abubaker J., Jun J., Allen J.W., Brouillette J., Douds D.D., Lammers P.J., Shachar-Hill Y. (2003). Carbon Export from Arbuscular Mycorrhizal Roots Involves the Translocation of Carbohydrate as Well as Lipid. Plant Physiol..

[24] Rozpądek P., Wężowicz K., Nosek M., Ważny R., Tokarz K., Lembicz M., Miszalski Z., Turnau K. (2015). The Fungal Endophyte Epichloë Typhina Improves Photosynthesis Efficiency of Its Host Orchard Grass (*Dactylis glomerata*). Planta.

[25] Baum M., Von Korff M., Guo P., Lakew B., Hamwieh A., Lababidi S., Udupa S.M., Sayed H., Choumane W., Grando S., Varshney R.K., Tuberosa R. (2007). Molecular Approaches and Breeding Strategies for Drought Tolerance in Barley. Genomics-Assisted Crop Improvement: Vol 2: Genomics Applications in Crops.

[26] Sabagh A.E., Hossain A., Islam M.S., Barutcular C., Hussain S., Hasanuzzaman M., Akram T., Mubeen M., Nasim W., Fahad S. (2019). Drought and Salinity Stresses in Barley: Consequences and Mitigation Strategies. Aust. J. Crop Sci..

[27] Zulfiqar F., Hancock J.T. (2020). Hydrogen sulfide in horticulture: Emerging roles in the era of climate change. Plant Physiol. Biochem..

[28] O’Donovan J.T., Turkington T.K., Edney M.J., Clayton G.W., McKenzie R.H., Juskiw P.E., Lafond G.P., Grant C.A., Brandt S., Harker K.N. (2011). Seeding Rate, Nitrogen Rate, and Cultivar Effects on Malting Barley Production. Agron. J..

[29] Asghar M.G., Bashir A., Fahad S., Saud S., Chen Y., Wu C., Wang D. (2020). Protagonist of Mineral Nutrients in Drought Stress Tolerance of Field Crops. Abiotic Stress in Plants.

[30] Sehar S., Adil M.F., Zeeshan M., Holford P., Cao F., Wu F., Wang Y. (2021). Mechanistic Insights into Potassium-Conferred Drought Stress Tolerance in Cultivated and Tibetan Wild Barley: Differential Osmoregulation, Nutrient Retention, Secondary Metabolism and Antioxidative Defense Capacity. Int. J. Mol. Sci..

[31] Andersen M.N., Jensen C.R., Lösch R. (1992). The Interaction Effects of Potassium and Drought in Field-Grown Barley. II. Nutrient Relations, Tissue Water Content and Morphological Development. Acta Agric. Scand. B Soil Plant Sci..

[32] Jones C.A., Jacobsen J.S., Wraith J.M. (2005). Response of Malt Barley to Phosphorus Fertilization Under Drought Conditions. J. Plant Nutr..

[33] Tavakol E., Jákli B., Cakmak I., Dittert K., Senbayram M. (2022). Optimization of Potassium Supply under Osmotic Stress Mitigates Oxidative Damage in Barley. Plants.

[34] Askarnejad M.R., Soleymani A., Javanmard H.R. (2021). Barley (*Hordeum vulgare* L.) Physiology Including Nutrient Uptake Affected by Plant Growth Regulators under Field Drought Conditions. J. Plant Nutr..

[35] Ragel P., Raddatz N., Leidi E.O., Quintero F.J., Pardo J.M. (2019). Regulation of K+ Nutrition in Plants. Front. Plant Sci..

[36] Zulfiqar F., Nafees M., Darras A., Shaukat N., Chen J., Ferrante A., Zaid A., Latif N., Raza A., Siddique K.H. (2022). Pre-harvest potassium foliar application improves yield, vase life and overall postharvest quality of cut gladiolus inflorescences. Postharvest Biol. Technol..

[37] Wójcik-Jagła M., Rapacz M., Tyrka M., Kościelniak J., Crissy K., Żmuda K. (2013). Comparative QTL Analysis of Early Short-Time Drought Tolerance in Polish Fodder and Malting Spring Barleys. Theor. Appl. Genet..

[38] Barnabás B., Jäger K., Fehér A. (2008). The Effect of Drought and Heat Stress on Reproductive Processes in Cereals. Plant Cell Environ..

[39] Kolář P., Trnka M., Brázdil R., Hlavinka P. (2014). Influence of Climatic Factors on the Low Yields of Spring Barley and Winter Wheat in Southern Moravia (Czech Republic) during the 1961–2007 Period. Theor. Appl. Climatol..

[40] Khosravinejad F., Heydari R., Farboodnia T. (2009). Effect of Salinity on Organic Solutes Contents in Barley. Pak. J. Biol. Sci..

[41] Munjonji L., Ayisi K.K., Vandewalle B., Haesaert G., Boeckx P., Leal Filho W., Belay S., Kalangu J., Menas W., Munishi P., Musiyiwa K. (2017). Carbon Isotope Discrimination as a Surrogate of Grain Yield in Drought Stressed Triticale. Climate Change Adaptation in Africa. Climate Change Management.

[42] Cernusak L.A., Ubierna N., Winter K., Holtum J.A., Marshall J.D., Farquhar G.D. (2013). Environmental and Physiological Determinants of Carbon Isotope Discrimination in Terrestrial Plants. New Phytol..

[43] Chen J., Chang S.X., Anyia A.O. (2011). The Physiology and Stability of Leaf Carbon Isotope Discrimination as a Measure of Water-Use Efficiency in Barley on the Canadian Prairies. J. Agron. Crop Sci..

[44] Schelling K., Born K., Weissteiner C., Kühbauch W. (2003). Relationships between Yield and Quality Parameters of Malting Barley (*Hordeum vulgare* L.) and Phenological and Meteorological Data. J. Agron. Crop Sci..

[45] Bütehorn B., Rhody D., Franken P. (2000). Isolation and Characterisation of Pitef1 Encoding the Translation Elongation Factor EF-1α of the Root Endophyte *Piriformospora indica*. Plant Biol..

[46] Lancashire P.D., Bleiholder H., Boom T.V.D., Langelüddeke P., Stauss R., Weber E., Witzenberger A. (1991). A Uniform Decimal Code for Growth Stages of Crops and Weeds. Ann. Appl. Biol..

[47] DuBois M., Gilles K.A., Hamilton J.K., Rebers P.A., Smith F. (1956). Colorimetric Method for Determination of Sugars and Related Substances. Anal. Chem..

[48] (1997). Native Starch—Determination of Starch Content—Ewers Polarimetric Method.

[49] Klem K., Rajsnerová P., Novotná K., Mìša P., Křen J. (2014). Changes in Vertical Distribution of Spectral Reflectance within Spring Barley Canopy as an Indicator of Nitrogen Nutrition, Canopy Structure and Yield Parameters. Agriculture (Pol’nohospodárstvo).

[50] Kaczmarek M., Fedorowicz-Strońska O., Głowacka K., Waśkiewicz A., Sadowski J. (2017). CaCl_2_ treatment improves drought stress tolerance in barley (*Hordeum vulgare* L.). Acta Physiol. Plant..

[51] Sazegari S., Zinati Z., Tahmasebi A. (2020). Dynamic transcriptomic analysis uncovers key genes and mechanisms involved in seed priming-induced tolerance to drought in barley. Gene Rep..

[52] Daszkowska-Golec A., Collin A., Sitko K., Janiak A., Kalaji H.M., Szarejko I. (2019). Genetic and physiological dissection of photosynthesis in barley exposed to drought stress. Int. J. Mol. Sci..

[53] de Mezer M., Turska-Taraska A., Kaczmarek Z., Glowacka K., Swarcewicz B., Rorat T. (2014). Differential physiological and molecular response of barley genotypes to water deficit. Plant Physiol. Biochem..

[54] Kirby E.J.M. (1988). Analysis of leaf, stem and ear growth in wheat from terminal spikelet stage to anthesis. Field Crops Res..

[55] Waddington S.R., Cartwright P.M., Wall P.C. (1983). A quantitative scale of spike initial and pistil development in barley and wheat. Ann. Bot..

[56] Gómez J.F., Wilson Z.A. (2012). Non-destructive staging of barley reproductive development for molecular analysis based upon external morphology. J. Exp. Bot..

[57] Boussora F., Allam M., Guasmi F., Ferchichi A., Rutten T., Hansson M., Helmy M.Y., Börner A. (2019). Spike developmental stages and ABA role in spikelet primordia abortion contribute to the final yield in barley (*Hordeum vulgare* L.). Bot. Stud..

[58] Hu Y., Barmeier G., Schmidhalter U. (2021). Genetic Variation in Grain Yield and Quality Traits of Spring Malting Barley. Agronomy.

[59] Hussain S., Ali B., Saqib M., Shanker A.K., Shanker C., Anand A., Maheswari M. (2022). Chapter 15—Seed Priming to Enhance Salt and Drought Stress Tolerance in Plants: Advances and Prospects. Climate Change and Crop Stress.

[60] Tabassum T., Ahmad R., Farooq M., Basra S.M.A. (2018). Improving the Drought Tolerance in Barley by Osmopriming and Biopriming. Int. J. Agric. Biol..

[61] Abideen Z., Cardinale M., Zulfiqar F., Koyro H.W., Rasool S.G., Hessini K., Darbali W., Zhao F., Siddique K.H. (2022). Seed endophyte bacteria enhance drought stress tolerance in *Hordeum vulgare* by regulating, physiological characteristics, antioxidants and minerals uptake. Front. Plant Sci..

