# Supplementary material for: Endophyte Inoculation and Elevated Potassium Supply on Productivity, Growth and Physiological Parameters of Spring Barley (Hordeum vulgare L.) Genotypes over Contrasting Seasons
Source: Plants (Basel). 2024 Apr 22;13(8):1168. doi: 10.3390/plants13081168 (PMC11054443; doi:10.3390/plants13081168)
Supplement: Supplementary file 1 [file plants-13-01168-s001.zip › plants-2892452-supplementary.pdf]

plants

MDPI

Endophyte Inoculation and Elevated Potassium Supply on Productivity, Growth and Physiological Parameters of Spring Barley (*Hordeum vulgare* L.) Genotypes Over Contrasting Seasons

Dominik Bleša, Pavel Matušinský, Milan Baláž, Zdeněk Nesvadba and Marta Zavřelová

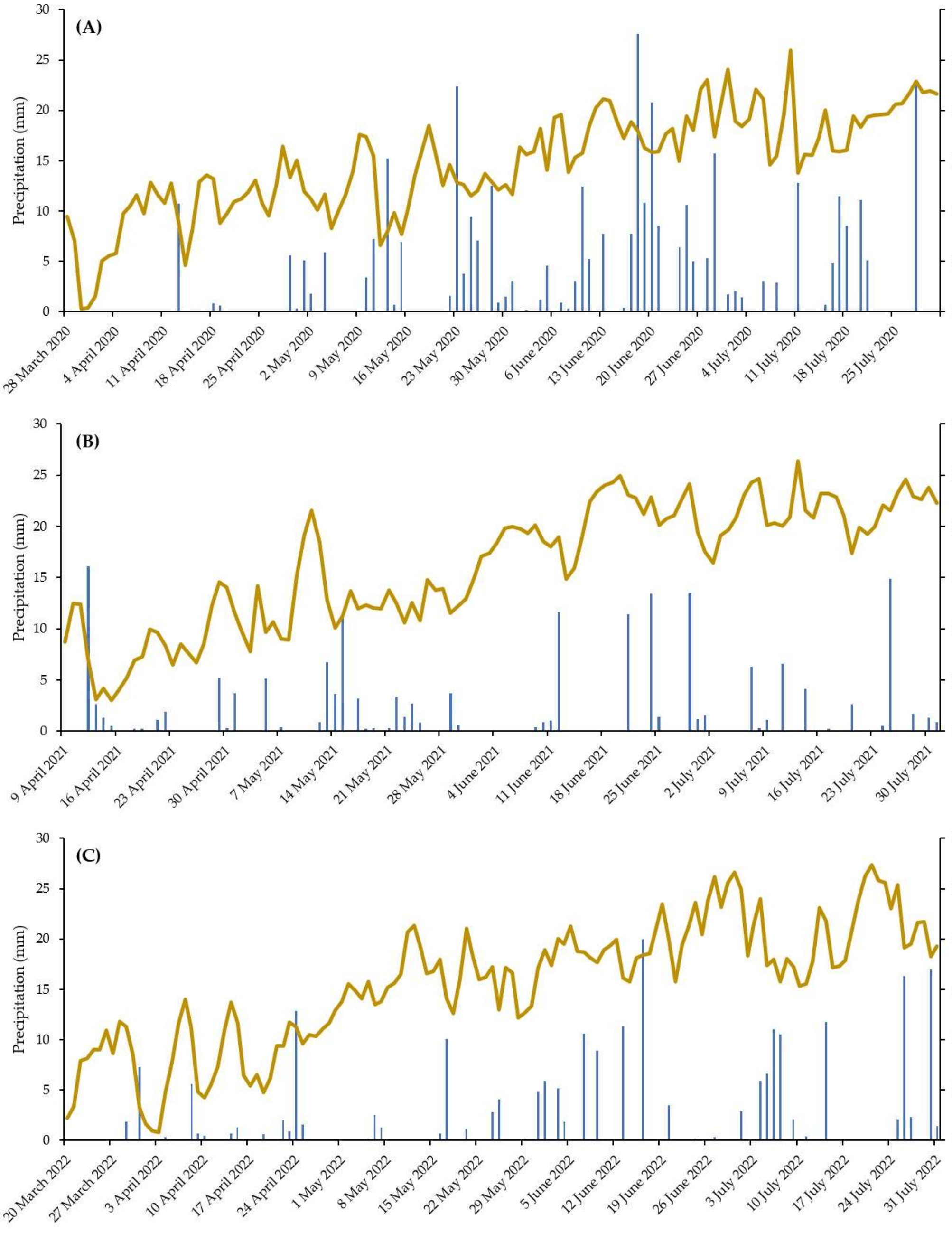

Figure S1. Mean temperatures and precipitation in growing seasons (**A**) 2020; (**B**) 2021; (**C**) 2022.

Table S1. The nutrient content in the soil taken at the experimental site before experiments.

Season	P (mg·kg-1)	K (mg·kg-1)	Ca (mg·kg-1)	Mg (mg·kg-1)	pH/KCl
2020	70	208	2553	285	5.04
2021	46	137	2355	253	5.27
2022	120	170	2890	153	6.44

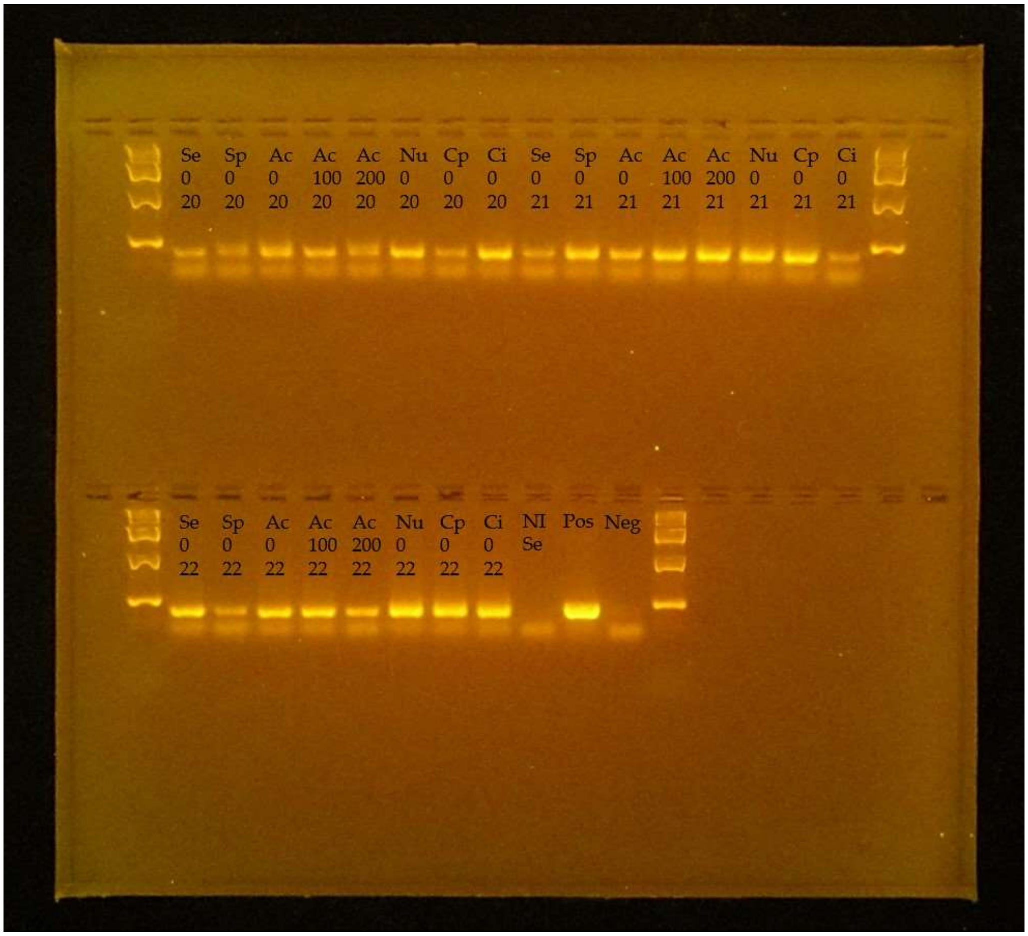

Figure S2. Electrophoresis gel of the PCR products using *Serendipita indica*-specific primers. Samples were isolated from barley roots in seasons 2020–2022. Se – Sebastian, Sp – Spitfire, Ac – Accordine, Nu – Nutans Afganistan, Cp – CPI 18197, Ci – CI 6388 genotypes; 0, 100, 200 – kg/ha potassium supply; 20 – season 2020, 21 – season 2021, 22 – season 2022; NI – not inoculated; Pos – positive control using fungal DNA, Neg – negative control.

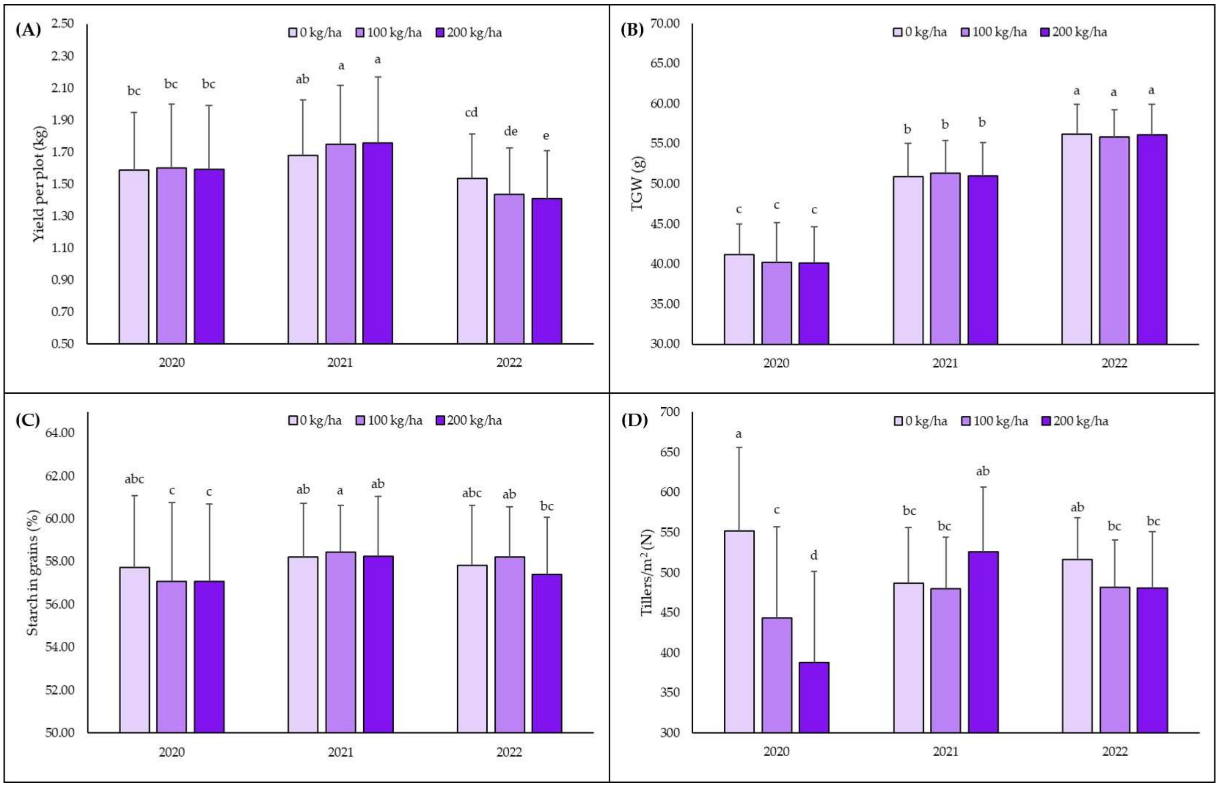

Figure S3. Interaction of fertilization and season on productive parameters and tillering. (**A**) yield per plot; (**B**) thousand grains weight; (**C**) starch in grains; (**D**) tillers. Columns represent means, bars *SD* (*n* = 30) followed by the same letter if there was no statistical difference according to Tukey0.05 test.

Table S2. Interaction of fertilization, inoculation and season factors on the thousand grains weight and plant height. Treatment is in format fertilization (0, 100, or 200 kg of potassium per ha) × inoculation (NI – not inoculated, *S. indica* – *Serendipita indica*) × season (2020–2022). Data represent means ± *SD* (*n* = 12 for *S. indica*; *n* = 18 for NI) followed by the same letter if there was no statistical difference according to Tukey0.05 test for unequal sample sizes.

Treatment	TGW (g)	Plant height (cm)
0×NI×2020	40.56 ± 3.82 cd	84.8 ± 6.5 a
0×NI×2021	50.91 ± 3.85 b	75.3 ± 7.5 cd
0×NI×2022	56.14 ± 3.80 a	68.1 ± 6.6 f
0×*S. indica*×2020	41.98 ± 3.86 c	82.0 ± 8.8 ab
0×*S. indica*×2021	50.80 ± 4.77 b	75.3 ± 8.4 cde
0×*S. indica*×2022	56.32 ± 3.69 a	70.5 ± 6.8 cdef
100×NI×2020	40.22 ± 4.96 cd	84.9 ± 9.8 a
100×NI×2021	51.24 ± 4.44 b	76.2 ± 6.6 bcd
100×NI×2022	55.88 ± 3.37 a	71.0 ± 8.5 cdef
100×*S. indica*×2020	40.13 ± 5.14 cd	88.4 ± 7.5 a
100×*S. indica*×2021	51.34 ± 3.80 b	75.5 ± 7.0 bcd
100×*S. indica*×2022	55.88 ± 3.51 a	68.5 ± 7.9 f
200×NI×2020	40.69 ± 4.97 cd	86.3 ± 7.2 a
200×NI×2021	50.73 ± 4.09 b	76.4 ± 5.7 bc
200×NI×2022	55.98 ± 4.00 a	68.6 ± 8.4 f
200×*S. indica*×2020	39.27 ± 3.81 d	88.0 ± 8.3 a

200×*S. indica*×2021	51.33 ± 4.43 b	76.2 ± 5.9 bcd
200×*S. indica*×2022	56.34 ± 3.65 a	69.5 ± 10.0 ef

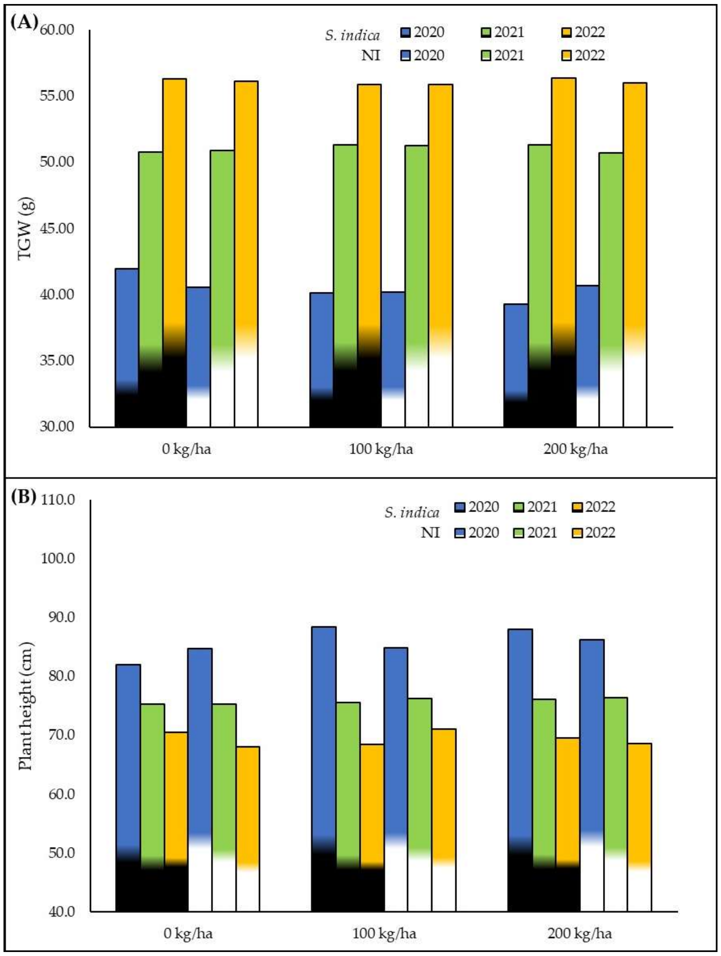

Figure S4. Interaction of fertilization, inoculation, and season factors on the (**A**) thousand grains weight (TGW) and (**B**) plant height. Fertilization doses 0, 100, or 200 kg of potassium per ha, season (2020–2022); and inoculation (NI – not inoculated, *S. indica* – *Serendipita indica*). Data represent means (*n* = 12 for *S. indica*; *n* = 18 for NI). Statistical significance and *SD* values are shown in the Supplementary Table 2.

Table S3. Interaction of genotype, fertilization and season factors on the tillering, plant height and carbon isotope signature (δ^{13}C). Treatment is in format genotype × fertilization (0, 100, or 200 kg of potassium per ha) × season (2020–2022). Data represent means ± *SD* (*n* = 5) followed by the same letter if there was no statistical difference according to Tukey$_{0.05}$ test.

Treatment	Tillers/m^2 (N)	Plant height (cm)	δ^{13}C (‰)
Sebastian×0×2020	608 ± 74 ab	75.4 ± 2.1 defghijklmnop	-29.8 ± 0.3 lmnopqrs
Sebastian×0×2021	610 ± 21 ab	61.6 ± 2.9 stuv	-29.1 ± 0.1 ghijklmno
Sebastian×0×2022	535 ± 75 abcdefgh	60.8 ± 1.9 tuv	-27.6 ± 0.2 de
Sebastian×100×2020	602 ± 56 abc	80.6 ± 3.8 bcdefghijkl	-30.2 ± 0.5 s
Sebastian×100×2021	573 ± 27 abcd	63.8 ± 3.0 rstuv	-29.1 ± 0.4 ghijklmno
Sebastian×100×2022	558 ± 80 abcdef	58.0 ± 3.5 v	-27.8 ± 0.2 ef
Sebastian×200×2020	486 ± 120 bcdefghijk	78.6 ± 1.3 bcdefghijklm	-30.3 ± 0.5 s
Sebastian×200×2021	642 ± 14 a	65.8 ± 0.8 opqrstuv	-28.9 ± 0.0 ghijkl

Sebastian×200×2022	568 ± 41 [abcd]	58.6 ± 2.6 [uv]	-27.7 ± 0.2 [de]
Spitfire×0×2020	568 ± 144 [abcd]	85.6 ± 6.4 [abcdef]	-30.0 ± 0.4 [qrs]
Spitfire×0×2021	480 ± 33 [bcdefghijkl]	72.0 ± 2.1 [ijklmnopqr]	-29.2 ± 0.2 [ghijklmnop]
Spitfire×0×2022	530 ± 24 [abcdefgh]	65.0 ± 3.2 [pqrstuv]	-27.3 ± 0.3 [cde]
Spitfire×100×2020	430 ± 91 [defghijklm]	82.4 ± 3.4 [bcdefghij]	-30.1 ± 0.3 [rs]
Spitfire×100×2021	529 ± 42 [abcdefgh]	74.4 ± 2.3 [efghijklmnopq]	-29.0 ± 0.2 [ghijklmn]
Spitfire×100×2022	473 ± 33 [bcdefghijklm]	67.0 ± 5.4 [nopqrstuv]	-27.2 ± 0.4 [cde]
Spitfire×200×2020	393 ± 57 [ghijklm]	86.0 ± 3.9 [abcde]	-30.1 ± 0.3 [rs]
Spitfire×200×2021	533 ± 28 [abcdefgh]	80.0 ± 2.0 [bcdefghijkl]	-29.0 ± 0.2 [ghijklmn]
Spitfire×200×2022	473 ± 63 [bcdefghijklm]	64.8 ± 3.6 [qrstuv]	-27.4 ± 0.4 [cde]
Accordine×0×2020	538 ± 116 [abcdefgh]	86.2 ± 3.0 [abcde]	-29.8 ± 0.3 [nopqrs]
Accordine×0×2021	508 ± 21 [abcdefghi]	81.4 ± 2.2 [bcdefghijk]	-28.5 ± 0.3 [fg]
Accordine×0×2022	496 ± 43 [abcdefghi]	67.2 ± 3.1 [nopqrstuv]	-27.4 ± 0.3 [cde]
Accordine×100×2020	542 ± 37 [abcdefg]	88.0 ± 6.3 [abc]	-29.8 ± 0.3 [mnopqrs]
Accordine×100×2021	494 ± 17 [abcdefghij]	81.0 ± 1.0 [bcdefghijk]	-28.8 ± 0.4 [ghijk]
Accordine×100×2022	472 ± 28 [bcdefghijklm]	67.8 ± 2.3 [mnopqrstu]	-26.9 ± 0.2 [cd]
Accordine×200×2020	447 ± 98 [cdefghijklm]	90.0 ± 1.6 [ab]	-29.8 ± 0.2 [lmnopqrs]
Accordine×200×2021	572 ± 22 [abcd]	81.4 ± 1.7 [bcdefghijk]	-28.9 ± 0.1 [ghijk]
Accordine×200×2022	464 ± 81 [bcdefghijklm]	65.8 ± 4.1 [opqrstuv]	-27.0 ± 0.3 [cde]
Nutans Afganistan×0×2020	562 ± 69 [abcde]	90.0 ± 6.0 [ab]	-29.5 ± 0.3 [hijklmnopqrs]
Nutans Afganistan×0×2021	488 ± 21 [abcdefghijk]	83.2 ± 2.0 [bcdefghi]	-28.7 ± 0.1 [gh]
Nutans Afganistan×0×2022	500 ± 36 [abcdefghi]	75.6 ± 6.0 [defghijklmno]	-26.0 ± 0.4 [ab]
Nutans Afganistan×100×2020	320 ± 46 [m]	91.8 ± 14.1 [ab]	-29.6 ± 0.3 [klmnopqrs]
Nutans Afganistan×100×2021	418 ± 23 [defghijklm]	81.2 ± 1.6 [bcdefghijk]	-28.7 ± 0.2 [ghi]
Nutans Afganistan×100×2022	442 ± 35 [defghijklm]	83.2 ± 2.8 [bcdefghi]	-25.7 ± 0.8 [a]
Nutans Afganistan×200×2020	339 ± 133 [jklm]	98.4 ± 8.5 [a]	-29.3 ± 0.4 [ghijklmnopqr]
Nutans Afganistan×200×2021	385 ± 15 [hijklm]	74.4 ± 1.5 [efghijklmnopq]	-28.6 ± 0.3 [fg]
Nutans Afganistan×200×2022	442 ± 55 [defghijklm]	84.2 ± 5.9 [bcdefgh]	-26.0 ± 0.5 [ab]
CPI 18197×0×2020	489 ± 82 [abcdefghijk]	77.2 ± 6.7 [cdefghijklmn]	-30.1 ± 0.1 [s]
CPI 18197×0×2021	405 ± 18 [fghijklm]	73.6 ± 2.5 [fghijklmnopqr]	-29.0 ± 0.2 [ghijklm]
CPI 18197×0×2022	532 ± 86 [abcdefgh]	72.4 ± 6.8 [ijklmnopqr]	-26.7 ± 0.2 [bc]
CPI 18197×100×2020	412 ± 65 [efghijklm]	86.0 ± 9.8 [abcde]	-29.5 ± 0.3 [ijklmnopqrs]
CPI 18197×100×2021	422 ± 39 [defghijklm]	73.6 ± 1.5 [fghijklmnopqr]	-29.3 ± 0.2 [ghijklmnopqr]
CPI 18197×100×2022	447 ± 52 [cdefghijklm]	72.6 ± 1.5 [hijklmnopqr]	-26.6 ± 0.5 [bc]
CPI 18197×200×2020	337 ± 72 [klm]	83.6 ± 5.0 [bcdefghi]	-29.6 ± 0.3 [jklmnopqrs]
CPI 18197×200×2021	514 ± 30 [abcdefgh]	75.4 ± 1.7 [defghijklmnop]	-29.2 ± 0.2 [ghijklmnopq]
CPI 18197×200×2022	442 ± 53 [defghijklm]	70.6 ± 3.6 [klmnopqrst]	-26.7 ± 0.2 [bc]
CI 6388×0×2020	548 ± 136 [abcdefg]	87.6 ± 7.2 [abcd]	-29.9 ± 0.2 [opqrs]
CI 6388×0×2021	429 ± 18 [defghijklm]	80.0 ± 1.2 [bcdefghijkl]	-28.8 ± 0.3 [ghijk]
CI 6388×0×2022	504 ± 27 [abcdefghi]	73.4 ± 3.8 [ghijklmnopqr]	-27.0 ± 0.3 [cde]
CI 6388×100×2020	354 ± 33 [ijklm]	89.0 ± 10.7 [abc]	-30.0 ± 0.6 [qrs]
CI 6388×100×2021	445 ± 10 [defghijklm]	81.6 ± 1.1 [bcdefghijk]	-28.8 ± 0.3 [ghij]
CI 6388×100×2022	499 ± 39 [abcdefghi]	71.4 ± 2.6 [jklmnopqrs]	-27.4 ± 0.3 [cde]
CI 6388×200×2020	325 ± 127 [lm]	85.2 ± 4.4 [abcdefg]	-30.0 ± 0.4 [pqrs]
CI 6388×200×2021	508 ± 19 [abcdefghi]	81.0 ± 1.9 [bcdefghijk]	-28.7 ± 0.3 [gh]
CI 6388×200×2022	496 ± 59 [abcdefghi]	69.8 ± 5.7 [lmnopqrst]	-27.3 ± 0.4 [cde]

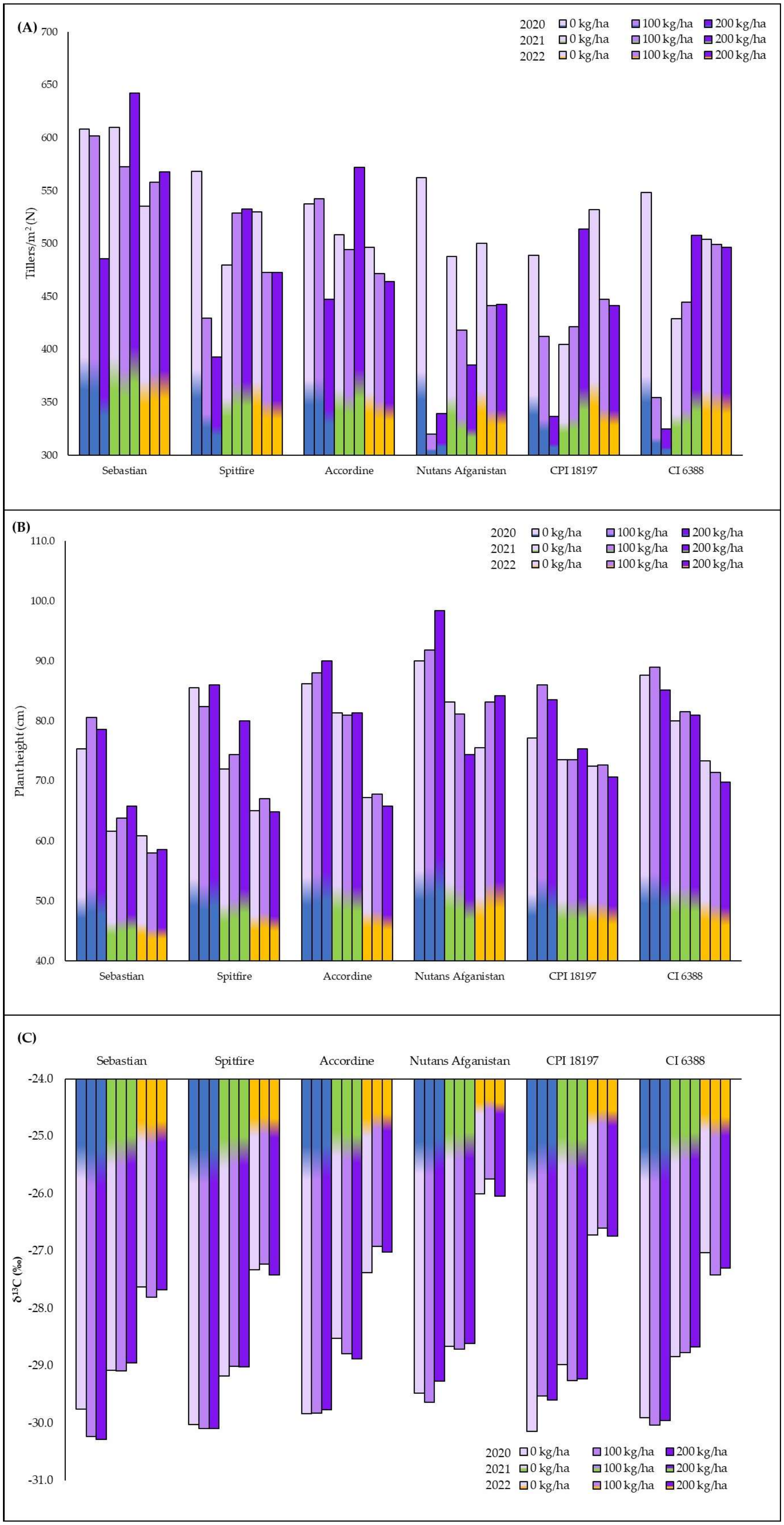
(A)
2020 0 kg/ha 100 kg/ha 200 kg/ha
2021 0 kg/ha 100 kg/ha 200 kg/ha
2022 0 kg/ha 100 kg/ha 200 kg/ha
Tillers/m² (N)
700
650
600
550
500
450
400
350
300
Sebastian
Spitfire
Accordine
Nutans Afganistan
CPI 18197
CI 6388
(B)
2020 0 kg/ha 100 kg/ha 200 kg/ha
2021 0 kg/ha 100 kg/ha 200 kg/ha
2022 0 kg/ha 100 kg/ha 200 kg/ha
Plant height (cm)
110.0
100.0
90.0
80.0
70.0
60.0
50.0
40.0
Sebastian
Spitfire
Accordine
Nutans Afganistan
CPI 18197
CI 6388
(C)
Sebastian
Spitfire
Accordine
Nutans Afganistan
CPI 18197
CI 6388
δ¹³C (‰)
-24.0
-25.0
-26.0
-27.0
-28.0
-29.0
-30.0
-31.0
2020 0 kg/ha 100 kg/ha 200 kg/ha
2021 0 kg/ha 100 kg/ha 200 kg/ha
2022 0 kg/ha 100 kg/ha 200 kg/ha

Figure S5. Interaction of genotype, fertilization, and season factors on the (**A**) tillering; (**B**) plant height; and (**C**) carbon isotope signature ($\delta^{13}C$). Fertilization doses 0, 100, or 200 kg of potassium per ha, season (2020–2022). Data represent means (*n* = 5). Statistical significance and *SD* values are shown in the Supplementary Table 4.

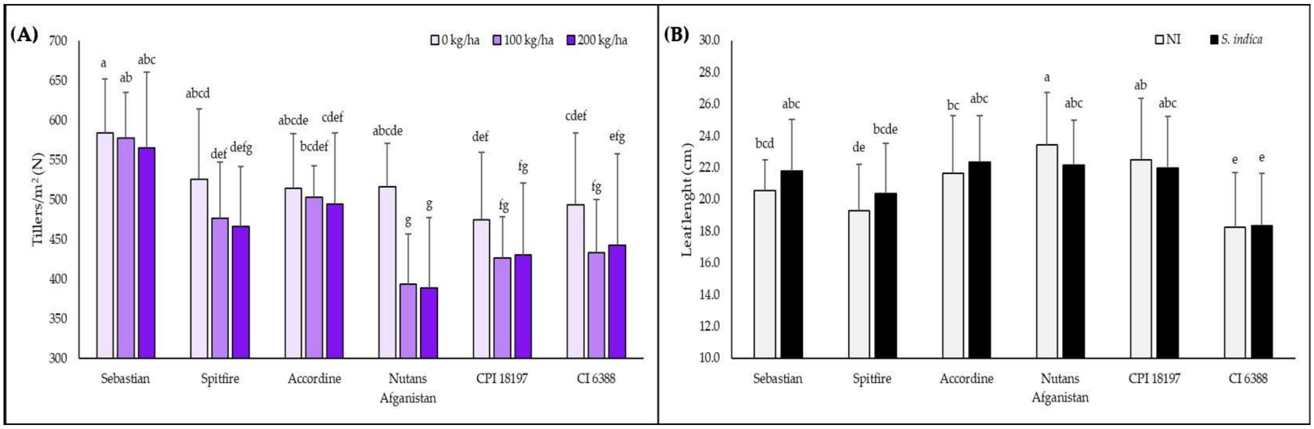

Figure S6. (**A**) Tillering of different genotypes in interaction with fertilization – columns represent means, bars *SD* (*n* = 15); (**B**) Leaf length in interaction of genotype and inoculation – columns represent means, bars *SD* (*n* = 18 for *S. indica*; *n* = 27 for NI); followed by the same letter if there was no statistical difference according to Tukey$_{0.05}$ test.

Table S4. Interaction of genotype, fertilization and inoculation factors on the tillering. Treatment is in format genotype × fertilization (0, 100, or 200 kg of potassium per ha) × inoculation (NI – not inoculated, *S. indica* – *Serendipita indica*). Data represent means ± *SD* (*n* = 6 for *S. indica*; *n* = 9 for NI) followed by the same letter if there was no statistical difference according to Tukey$_{0.05}$ test for unequal sample sizes.

Treatment	Tillers/m^2 (N)
Sebastian×0×NI	580 ± 63 ^{abc}
Sebastian×0×*S. indica*	591 ± 80 ^{abc}
Sebastian×100×NI	561 ± 36 ^{abcd}
Sebastian×100×*S. indica*	602 ± 77 ^{ab}
Sebastian×200×NI	586 ± 54 ^{abc}
Sebastian×200×*S. indica*	535 ± 137 ^{abcdefghi}
Spitfire×0×NI	551 ± 105 ^{abcde}
Spitfire×0×*S. indica*	488 ± 34 ^{abcdefghijk}
Spitfire×100×NI	477 ± 89 ^{abcdefghijk}
Spitfire×100×*S. indica*	478 ± 32 ^{abcdefghijk}
Spitfire×200×NI	452 ± 73 ^{defghijk}
Spitfire×200×*S. indica*	487 ± 82 ^{abcdefghijk}
Accordine×0×NI	540 ± 67 ^{abcdefg}
Accordine×0×*S. indica*	475 ± 56 ^{abcdefghijk}
Accordine×100×NI	506 ± 50 ^{abcdefghij}
Accordine×100×*S. indica*	499 ± 23 ^{abcdefghij}
Accordine×200×NI	510 ± 83 ^{abcdefghij}
Accordine×200×*S. indica*	470 ± 101 ^{abcdefghijk}
Nutans Afganistan×0×NI	522 ± 68 ^{abcdefghi}
Nutans Afganistan×0×*S. indica*	509 ± 28 ^{abcdefghij}
Nutans Afganistan×100×NI	402 ± 57 ^{hijk}

Nutans Afganistan×100×*S. indica*	380 ± 77 [jk]
Nutans Afganistan×200×NI	361 ± 102 [k]
Nutans Afganistan×200×*S. indica*	431 ± 42 [defghijk]
CPI 18197×0×NI	468 ± 83 [abcdefghijk]
CPI 18197×0×*S. indica*	486 ± 94 [abcdefghijk]
CPI 18197×100×NI	441 ± 56 [defghijk]
CPI 18197×100×*S. indica*	405 ± 39 [ghijk]
CPI 18197×200×NI	451 ± 92 [defghijk]
CPI 18197×200×*S. indica*	401 ± 87 [hijk]
CI 6388×0×NI	456 ± 53 [bcdefghijk]
CI 6388×0×*S. indica*	551 ± 108 [abcdef]
CI 6388×100×NI	430 ± 78 [fghijk]
CI 6388×100×*S. indica*	437 ± 56 [defghijk]
CI 6388×200×NI	461 ± 102 [bcdefghijk]
CI 6388×200×*S. indica*	416 ± 137 [fghijk]

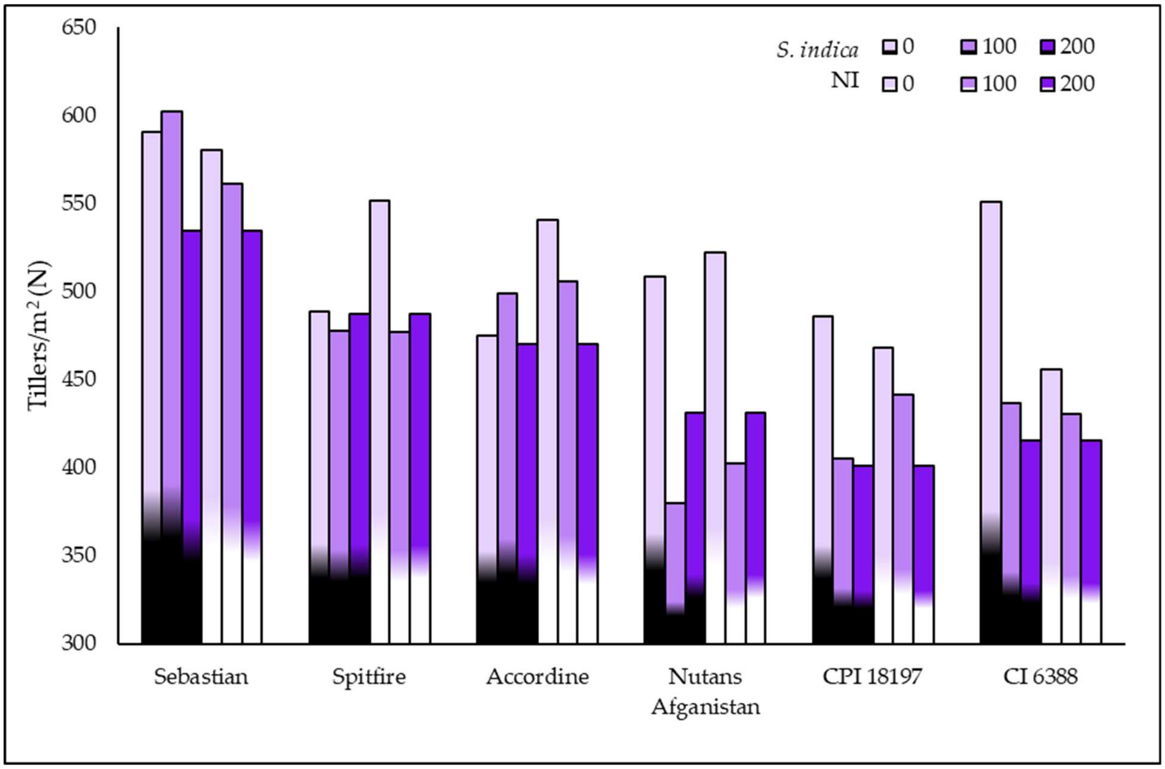

Figure S7. Interaction of genotype, fertilization, and inoculation factors on the tillering. Fertilization doses 0, 100, or 200 kg of potassium per ha, inoculation (NI – not inoculated, *S. indica* – *Serendipita indica*). Data represent means (n = 6 for *S. indica*; n = 9 for NI). Statistical significance and *SD* values are shown in the Supplementary Table 3.
